# Supplementary material for: Structural Basis for Rab8a Recruitment of RILPL2 via LRRK2 Phosphorylation of Switch 2
Source: Structure. 2020 Apr 7;28(4):406–417.e6. doi: 10.1016/j.str.2020.01.005 (PMC7139218; doi:10.1016/j.str.2020.01.005)
Supplement: Document S1. Figures S1–S6 [file mmc1.pdf]

Structure, Volume *28*

Supplemental Information

Structural Basis for Rab8a Recruitment

of RILPL2 via LRRK2 Phosphorylation of Switch 2

Dieter Waschbüsch, Elena Purlyte, Prosenjit Pal, Emma McGrath, Dario R. Alessi, and Amir R. Khan

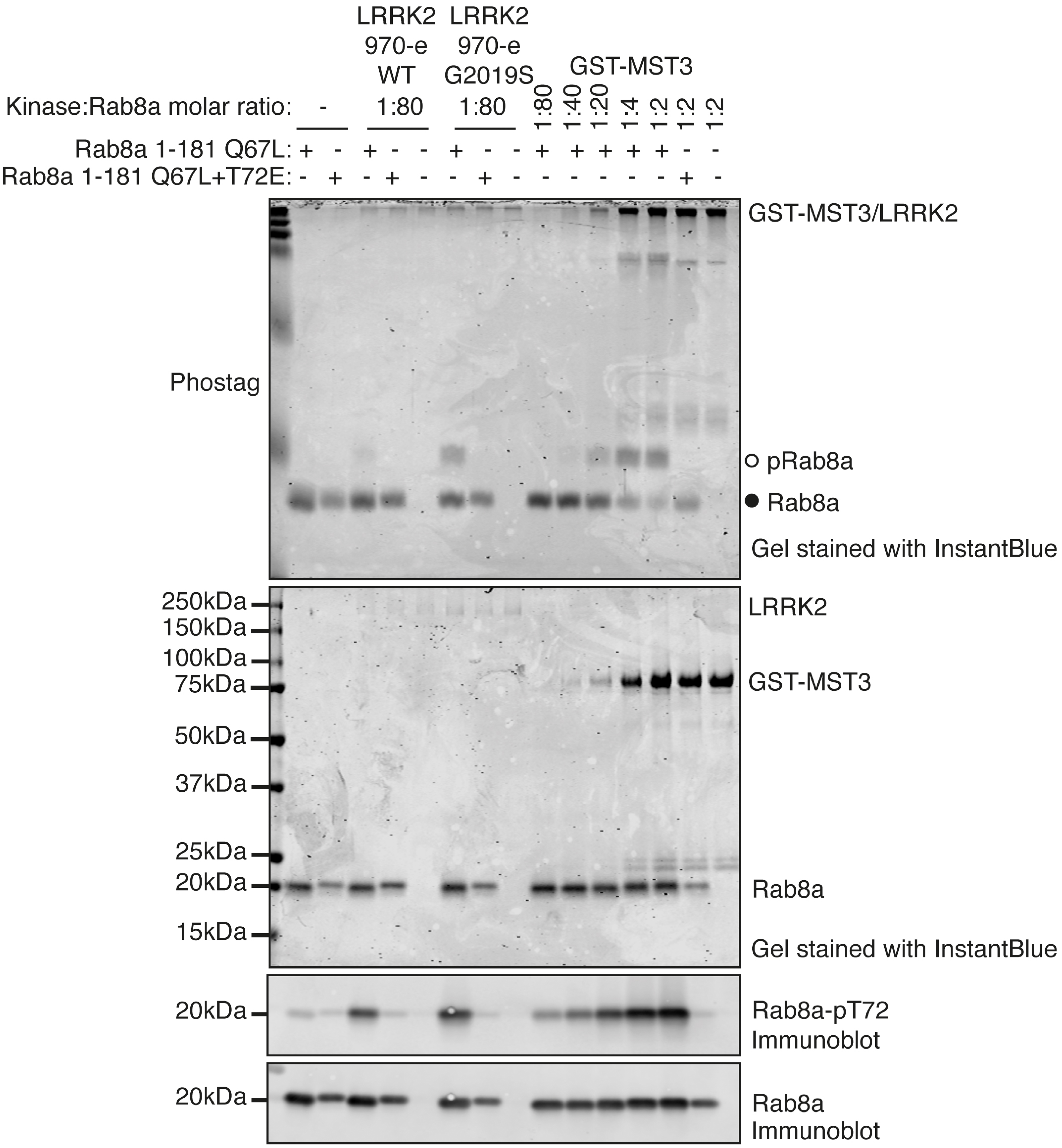

Figure S1, related to Figure 1: Phos-tag analysis of MST3 mediated Rab8a phosphorylation. Side by side analysis of wild type and G2019S insect cell expressed LRRK2 (residue 970-end) and full length insect cell expressed GST-MST3 mediated phosphorylation of recombinant Rab8a[Q67L, residues 1 to 181] or Rab8a[Q67L+T72E, residues 1 to 181]. Reactions were undertaken in the presence of 10 mM MgCl2 and 2 mM ATP for 3 hours at 37oC. Reactions were terminated by the addition of SDS sample buffer. Upper panel an aliquot of the reaction was analyzed by Phos-tag gel electrophoresis and protein visualized Coomassie Blue-staining. Bands corresponding to phosphorylated and non-phosphorylated Rab8a were marked with open (○) and closed (●) circles respectively. Lower panels samples were subjected to conventional gel electrophoresis and either stained with Coomassie (second panel) or subjected to immunoblot analysis using the LI-COR Odyssey CLx Western Blot imaging system with the indicated antibodies at 0.5-1 μg/mL concentration (two bottom panels). Similar results were obtained in at least two separate experiments.

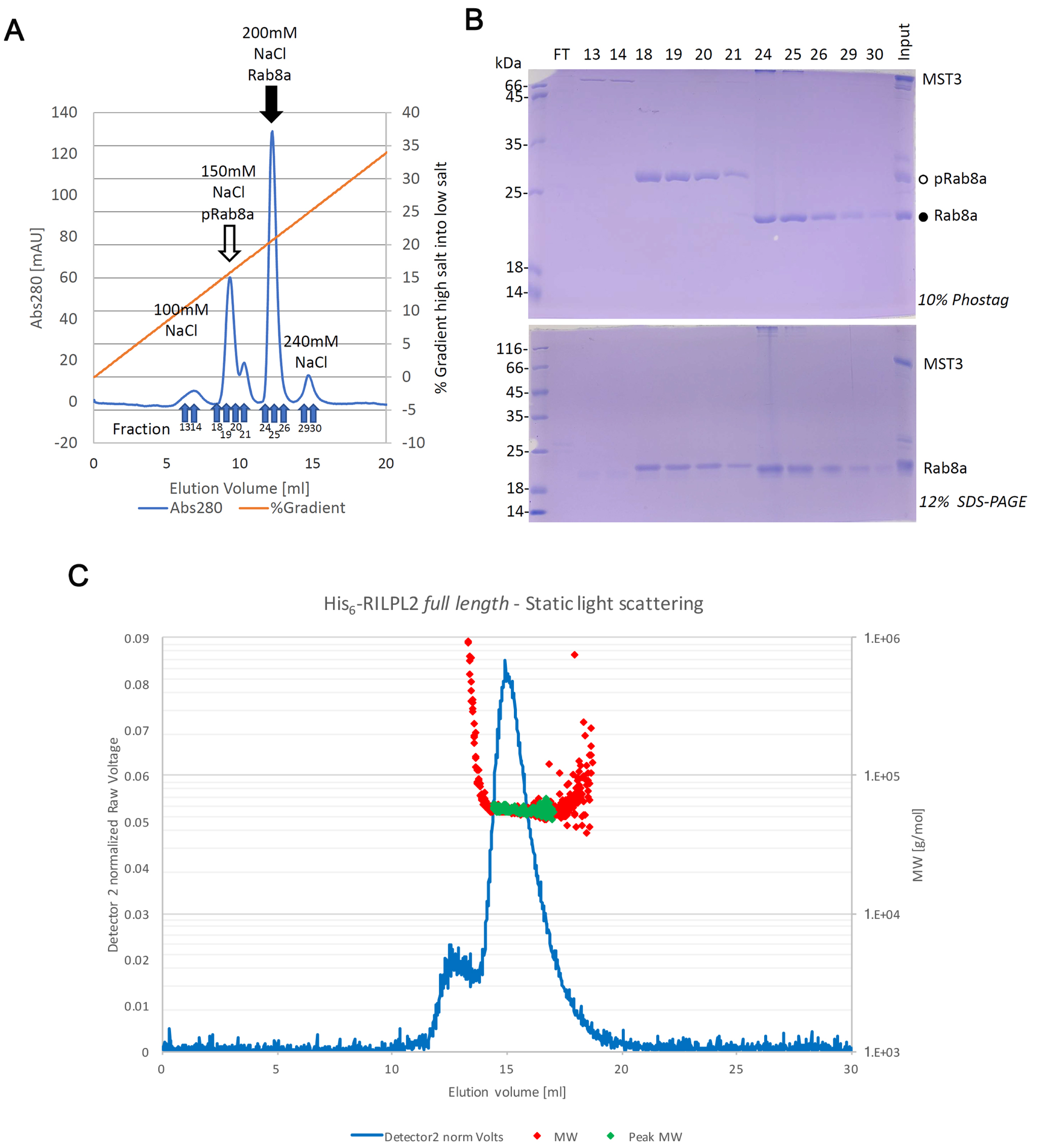

Figure S2, related to Figure 2: Purification of phosphorylated Rab8a. Following incubation of several mg of Rab8a1-181Q67L in the presence of GST-MST3 and ATP, phosphorylated Rab8a (pRab8a) was separated from the unphosphorylated protein by ion-exchange chromatography. A, Following dialysis in low salt buffer, reactants were loaded onto Mono-S cation exchange column (GE Healthcare) and eluted with a 50% gradient from low salt (10 mM) to high salt (1 M) buffered solution. During the salt gradient, 0.5 ml fractions were collected, and the two main peaks (arrows) corresponded to pRab8a and Rab8a. B, PhosTag gel (upper panel) and SDS-PAGE (lower panel) of the collected fractions. FT: unbound fraction flow through; Input: endpoint of phosphorylation reaction before loading the column. The PhosTag gel shows that the peak coming off at 150 mM NaCl contains the phosphorylated Rab8a. C, Static light scattering (SLS) analysis of full-length RILPL2. The monomer, which includes the polyhistidine tag, is 27kDa. The effector runs as a dimer in this experiment (55kDa) with the SLS instrument coupled to a Superdex 200 (10/300) gel filtration column. Detector 2 correspond to the refractive index, which is related to the protein concentration.

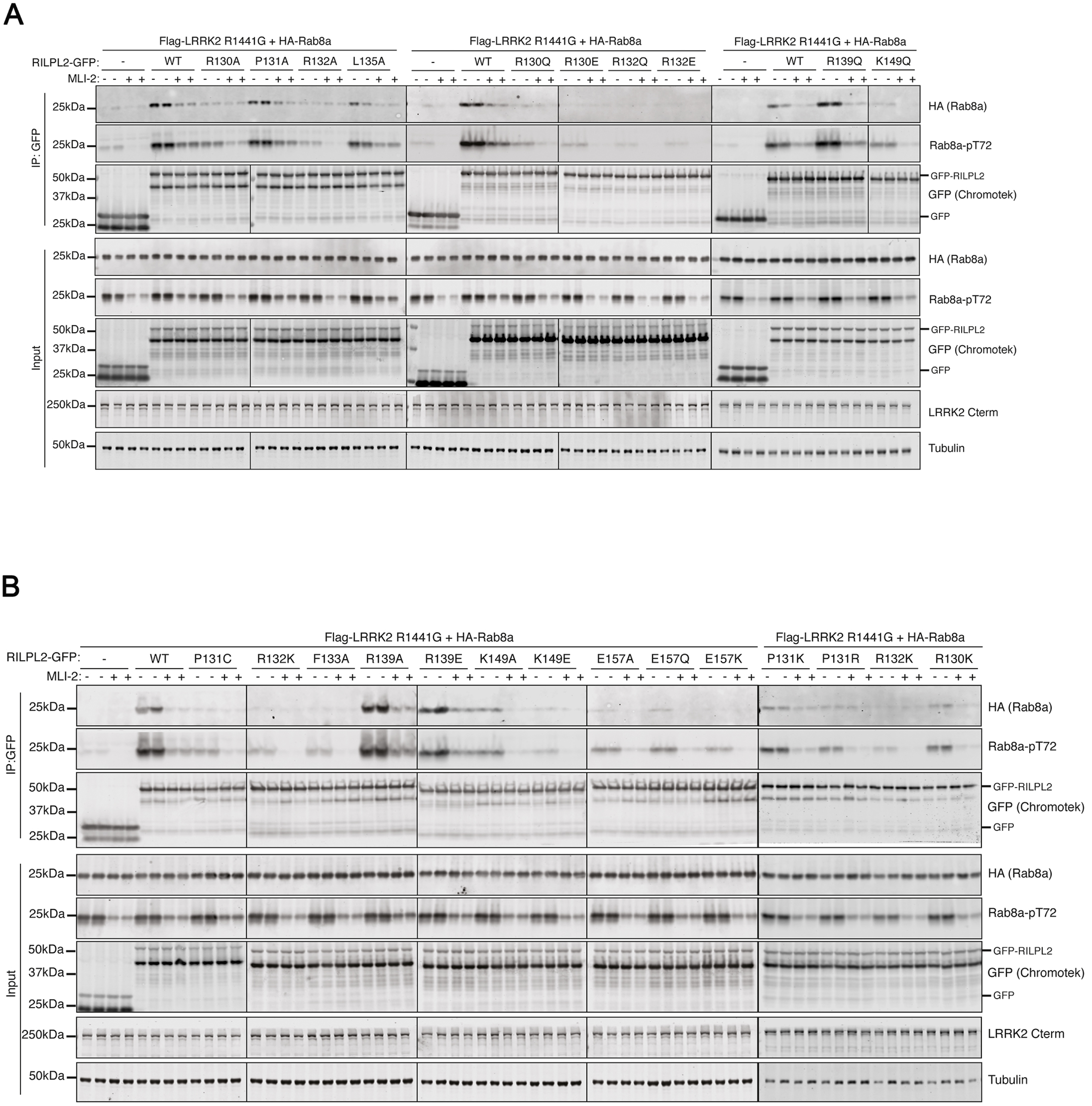

Figure S3, related to Figure 3: Figure legend same as for Figure 3.

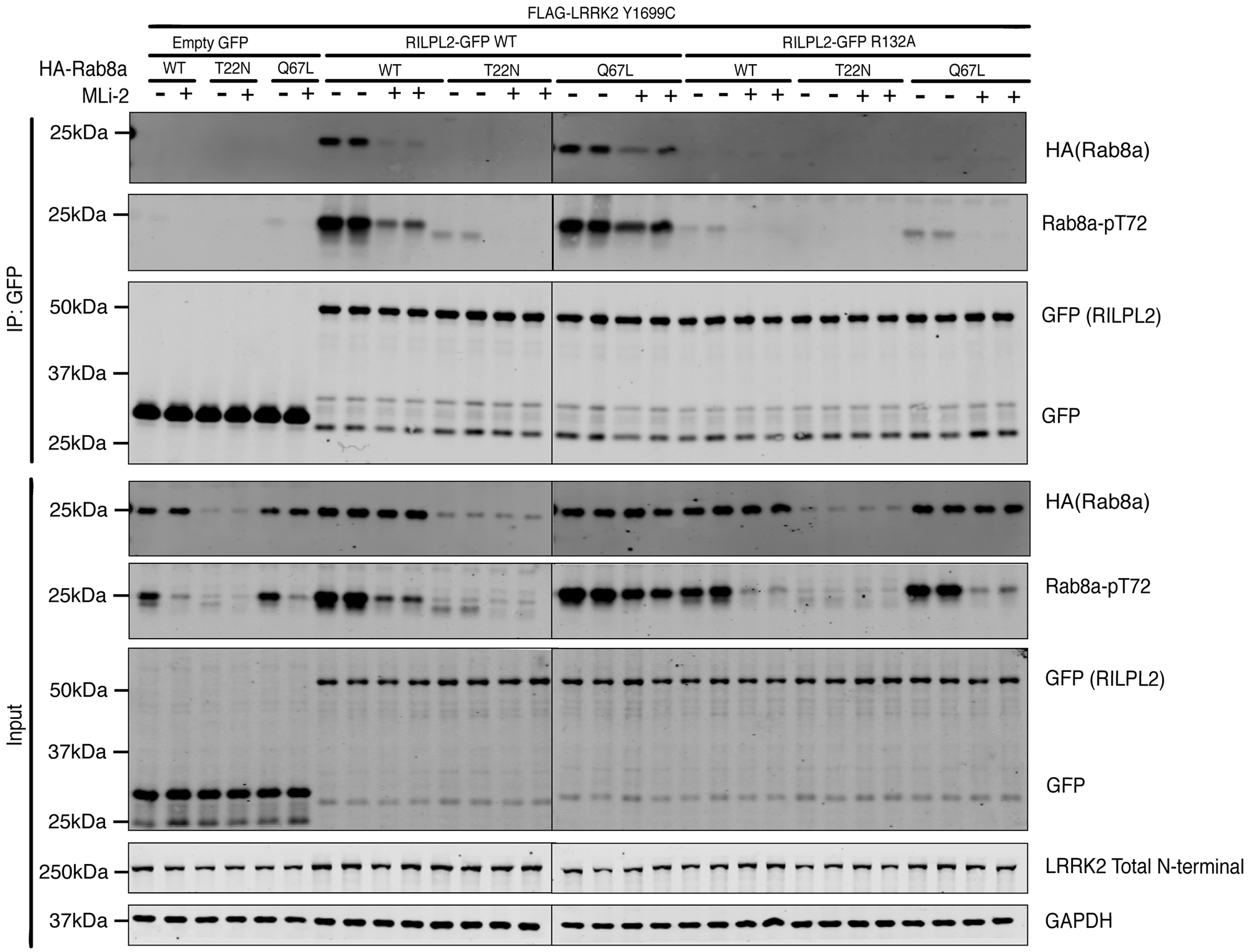

Figure S4, related to Figure 4: Same figure legend as Fig 4B

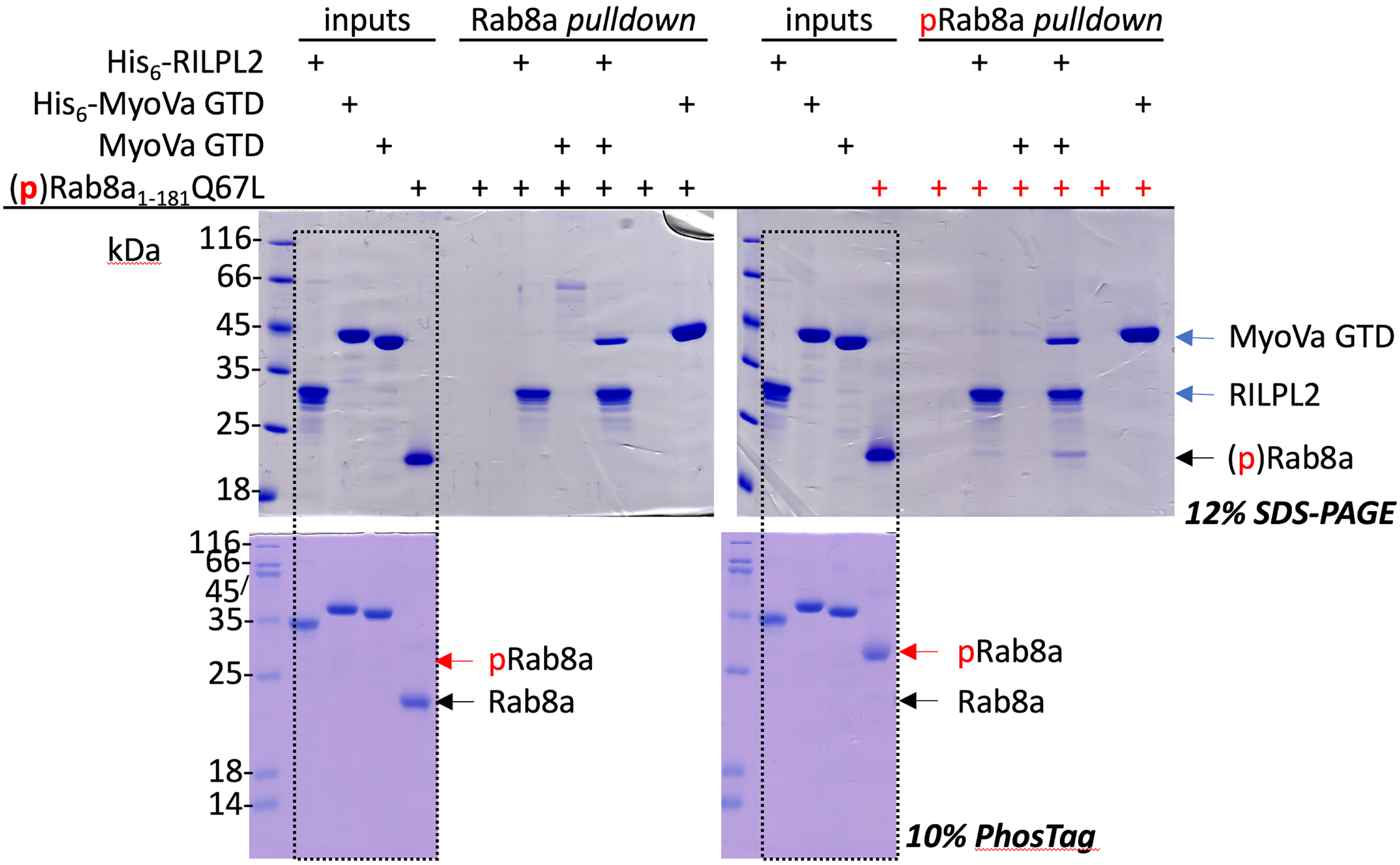

Figure S5, related to Figure 5A: Ni-NTA pulldowns of His6 tagged RILPL2 were performed using 2.5µM concentrations of bait and prey proteins. A control using pRab8a (red) and Rab8a pulldown using His6-tagged GTD of MyoVa confirms that there is no direct binding of either pRab8a or Rab8a to the GTD. Phostag gels of the input proteins are below the pulldowns.

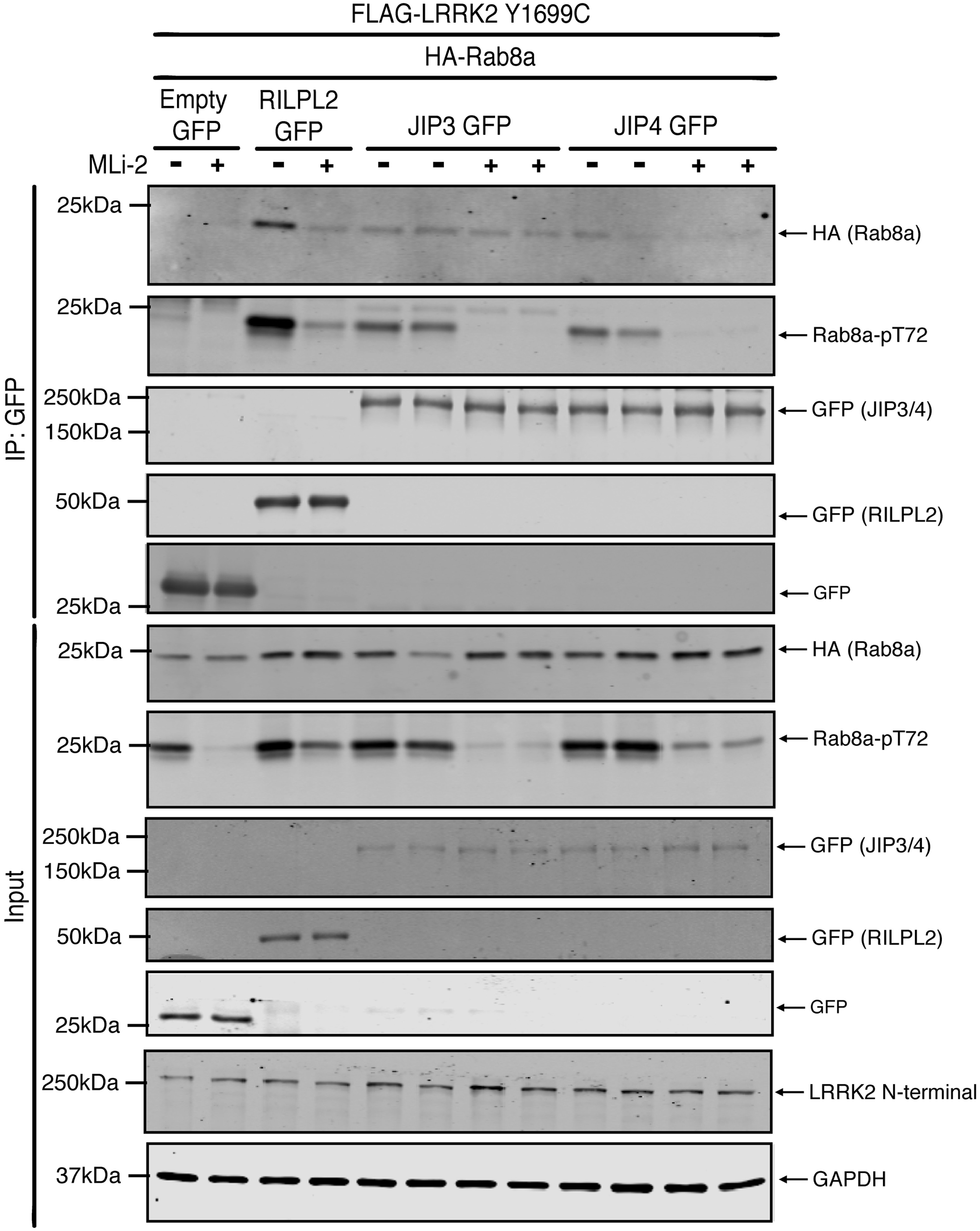

Figure S6, related to Figure 6A: JIP3 and JIP4 bind weakly to both Rab8a and LRRK2-phosphorylated Rab8a in cells. Same figure legend as Fig 6A.
