# Supplementary material for: Structural Basis for Rab8a Recruitment of RILPL2 via LRRK2 Phosphorylation of Switch 2
Source: Structure. 2020 Apr 7;28(4):406–417.e6. doi: 10.1016/j.str.2020.01.005 (PMC7139218; doi:10.1016/j.str.2020.01.005)
Supplement: Document S2. Article plus Supplemental Information [file mmc2.pdf]

Structure

Article

Structural Basis for Rab8a Recruitment of RILPL2 via LRRK2 Phosphorylation of Switch 2

Highlights

- Structure of LRRK2-phosphorylated Rab8a in complex with RH2 domain of effector RILPL2
- Phosphothreonine in switch 2 of Rab8a recognized by an arginine from RILPL2
- JIP3 and JIP4 have conserved arginine and bind to LRRK2-phosphorylated Rab10
- Conserved mode of phospho-Rab recognition by RH2 domain of effector proteins

Authors

Dieter Waschbüsch, Elena Purlyte, Prosenjit Pal, Emma McGrath, Dario R. Alessi, Amir R. Khan

Correspondence

amir.khan@tcd.ie or khan@crystal.harvard.edu

In Brief

LRRK2 is a serine/threonine kinase associated with Parkinson disease. LRRK2 phosphorylates Rab8a (pRab8a), which subsequently binds effector RILPL2 and regulates ciliogenesis. Waschbüsch et al. describe the structure of the pRab8a:RILPL2 complex and how phosphothreonine is recognized by an arginine from RILPL2. The structure reveals how LRRK2 assembles a trafficking complex.

Waschbüsch et al., 2020, Structure *28*, 406–417
April 7, 2020 Crown Copyright © 2020 Published by Elsevier Ltd.
https://doi.org/10.1016/j.str.2020.01.005

CellPress

Structural Basis for Rab8a Recruitment of RILPL2 via LRRK2 Phosphorylation of Switch 2

Dieter Waschbüsch,[1,4] Elena Purlyte,[3,4] Prosenjit Pal,[3] Emma McGrath,[1] Dario R. Alessi,[3] and Amir R. Khan[1,2,5,*]
[1]School of Biochemistry and Immunology, Trinity College, Dublin 2, Ireland
[2]Division of Newborn Medicine, Boston Children's Hospital, Boston, USA
[3]MRC Protein Phosphorylation and Ubiquitylation Unit, School of Life Sciences, University of Dundee, Dundee, UK
[4]These authors contributed equally
[5]Lead Contact
*Correspondence: amir.khan@tcd.ie or khan@crystal.harvard.edu
https://doi.org/10.1016/j.str.2020.01.005

SUMMARY

Rab8a is associated with the dynamic regulation of membrane protrusions in polarized cells. Rab8a is one of several Rab GTPases that are substrates of leucine-rich repeat kinase 2 (LRRK2), a serine/threonine kinase that is linked to Parkinson's disease. Rab8a is phosphorylated at T72 (pT72) in its switch 2 helix and recruits the phospho-specific effector RILPL2, which subsequently regulates ciliogenesis. Here, we report the crystal structure of phospho-Rab8a (pRab8a) in complex with the RH2 (RILP homology) domain of RILPL2. The complex is a heterotetramer with RILPL2 forming a central α-helical dimer that bridges two pRab8a molecules. The N termini of the α helices cross over, forming an X-shaped cap (X-cap) that orients Arg residues from RILPL2 toward pT72. X-cap residues critical for pRab8a binding are conserved in JIP3 and JIP4, which also interact with LRRK2-phosphorylated Rab10. We propose a general mode of recognition for phosphorylated Rab GTPases by this family of phospho-specific effectors.

INTRODUCTION

Parkinson's disease (PD) is a disorder of the CNS that manifests as a progressive degeneration of motor mobility, loss of balance, and tremors. Features of the pathology include loss of dopaminergic neurons in the midbrain and the presence of protein aggregates termed Lewy bodies, composed mainly of α-synuclein, in surviving neurons (Fahn, 2003). About 10% of cases have a genetic basis, with the most common gene being the leucine-rich repeat kinase 2 (LRRK2) (Schulte and Gasser, 2011). The gene product is a 2,527-residue (286 kDa) protein with multiple domains belonging to the ROCO family that is involved in regulation of autophagy, mitochondria, and Golgi dynamics (Roosen and Cookson, 2016). The kinase domain, located near the C terminus, phosphorylates itself and other proteins at serine/threonine residues (Greggio et al., 2008; Schulte and Gasser, 2011; Steger et al., 2016). Preceding the kinase domain, there is a Ras-like ROC domain (Ras of complex) followed in tandem by a COR domain (C-terminal of Ras). The ROC domain binds nucleotides (GTP/GDP) and is distantly related to the Rab family of small GTPases. The ROC-COR tandem domains regulate LRRK2 activity, and numerous missense mutations have been localized to these regulatory and kinase domains (Hui et al., 2018; Paisan-Ruiz et al., 2004; Zimprich et al., 2004). In addition to early onset forms of PD associated with autosomal dominant mutations, LRRK2 is also linked to late-onset sporadic cases of PD (Bardien et al., 2011).

Insight into LRRK2 functions has progressed significantly with the finding that a subset of small GTPases that include Rab8 and Rab10 are physiological substrates of the enzyme (Steger et al., 2016; West and Cookson, 2016). Rabs comprise the largest group (∼70 members) of the Ras superfamily, and they cycle between an active GTP bound and inactive GDP form to regulate membrane trafficking in eukaryotic cells (Hutagalung and Novick, 2011). The nucleotide-bound state of Rabs is regulated by GTPase-activating proteins (GAPs) and GTP/GDP exchange factors (GEFs), and active Rabs migrate to distinct sub-cellular compartments where they recruit cytosolic effector proteins. The "switch" regions of Rabs, termed switch 1 and 2, undergo local conformational changes that enable recruitment of GTP-specific effectors, which subsequently control processes, such as vesicle formation/fusion, motility, and other aspects of cell dynamics (Khan and Menetrey, 2013). LRRK2 phosphorylates Rab8a at T72 and Rab10 at T73, conserved threonine residues located on the α-helical switch 2 region. This post-translational modification modulates interactions between Rabs and their binding partners (Steger et al., 2016, 2017). For example, it inhibits interaction with Rab GDP dissociation inhibitors (GDIs) and Rabin-8, a GEF for Rab8a (Steger et al., 2016). LRRK2 phosphorylation of Rab8a and Rab10 also promoted interaction with two poorly studied scaffolding proteins termed RILPL1 (Rab interacting lysosomal protein-like 1) and RILPL2 (Steger et al., 2017), that were previously implicated in regulating ciliogenesis (Schaub and Stearns, 2013). RILPL1 and RILPL2 belong to the RILP family of effector proteins (Jordens et al., 2001). Unlike RILPL1 and RILPL2, RILP was not observed to interact with LRRK2 phosphorylated Rab8a or Rab10 (Steger et al., 2017); however, it is a known effector for Rab7a GTPase (Cantalupo et al., 2001). A recent study has suggested that RILP may bind more strongly to Rab7a phosphorylated at the equivalent site to LRRK2 by the related LRRK1 kinase (Hanafusa et al., 2019). Cellular studies

 Crown Copyright © 2020 Published by Elsevier Ltd.
This is an open access article under the CC BY license (http://creativecommons.org/licenses/by/4.0/).

Table 1. Crystallographic Data and Refinement Statistics

Data Collection	
Beamline	NECAT APS, 24-ID-E
Wavelength (Å)	0.9792
Space group	$P\ 2_1\ 2_1\ 2_1$
Cell dimensions	
a, b, c, (Å)	60.333, 71.509, 114.784
Resolution (Å)	53.41–1.767 (1.83–1.767)
Total reflections	324,590 (29,329)
Unique reflections	49,201 (4,719)
Completeness (%)	99.55 (96.33)
$\langle I/\sigma \rangle$	16.4 (1.69)
Multiplicity	6.6 (6.2)
R_{merge}	0.06877 (0.9488)
R_{meas}	0.07469 (1.036)
R_{pim}	0.02883 (0.4097)
$CC_{1/2}$	0.999 (0.649)
**Refinement**	
No. of reflections for R_{work}	49,193 (4,718)
No. of reflections for R_{free}	2,419 (241)
R_{work}	0.1789 (0.2882)
R_{free}	0.2105 (0.3049)
No. of non-hydrogen atoms	3,832
Macromolecules	3,401
Ligands	72
Solvent	359
Protein residues	416
RMSD	
Bond lengths (Å)	0.007
Bond angles (°)	0.91
Average overall B factor	36.54
Mean B factors (Å^2)	
Protein	35.84
Ligand	29.72
Water	44.53
Ramachandran analysis (%)	
Favored	97.24
Allowed	2.76
PDB:	6rir

Values in parentheses in the column on the right correspond to the statistics in the highest-resolution bin. RMSD, root-mean-square deviation. $R_{merge} = \Sigma_{hkl} \Sigma_j |I_{hkl,j} - \langle I_{hkl} \rangle| / \Sigma_{hkl} \Sigma_{jhkl,j}$. $R_{work} = \Sigma_{hkl}|F_{o,hkl} - F_{c,hkl}|/\Sigma_{hkl}F_{o,hkl}$.

have confirmed that LRRK2 blocks ciliogenesis by phosphorylating Rab8a and Rab10 and promoting RILPL1 interaction (Dhekne et al., 2018). RILPL1 and RILPL2 are homologous and were shown to interact with LRRK2-phosphorylated Rab8a and Rab10 via a C-terminal phospho-Rab binding domain that encompasses a conserved region of the protein. This region of the protein is also known as the RILP homology domain 2 (RH2) and encompasses residues 291–356 on RILPL1 and residues 130–201 on RILPL2. RILPL1 and RILPL2 also contain an N-terminal RH1 domain that binds to the globular tail domain (GTD) of myosin Va (Lise et al., 2009).

Upstream of the kinase, it has been shown that Rab29 recruits LRRK2 to the Golgi and activates the kinase, leading to an enhanced phosphorylation of substrate Rab GTPases, as well as increased autophosphorylation (Fujimoto et al., 2018; Liu et al., 2018; Purlyte et al., 2017). Rab32 is not a target for the kinase but it interacts with LRRK2 and regulates its sub-cellular localization (Waschbusch et al., 2014). These findings place LRRK2 at the center of a Rab signaling cascade that is key to understanding the molecular pathways that underpin PD. Recently, PPM1H has been identified as the phosphatase that counters LRRK2 activity via a phosphatase family small interfering RNA screen (Berndsen et al., 2019). PPM1H efficiently hydrolyses the phosphate from pRab8a and pRab10 substrates *in vitro* and in cells. Reversible phosphorylation provides a means of tuning the strength of interactions between Rab GTPases and their interacting proteins. Here, we describe the crystal structure of T72 phosphorylated Rab8a(GTP) in complex with a minimal phospho-Rab binding domain of RILPL2 at 1.8 Å resolution. The structure reveals that the phosphothreonine (pT72) is recognized by a conserved arginine from the RILP family of proteins. Moreover, RILPL2-related proteins JIP3 and JIP4 interact with pT73-Rab10 suggesting a general mechanism for phospho-specific recognition of effectors by Rab GTPases.

RESULTS

Overall Structure of the pRab8a:RILPL2 Complex

For these studies we utilized a mutant of the globular G-domain of Rab8a (Q67L, residues 1–181) that binds GTP constitutively. Descriptions of the *in vitro* kinase reaction and subsequent purification of pRab8a are described in Figures S1 and S2. Full-length RILPL2 complexes failed to crystallize, but the phospho-Rab binding domain of RILPL2 (residues 129–165) yielded crystals in complex with pRab8a. This region is the minimal RH2 motif with high sequence similarities to all members of the RILP effector family. Crystals of the complex diffract to 1.8 Å resolution (Table 1). The complex of pRab8a(GTP):RILPL2 is organized as a heterotetramer in the asymmetric unit (Figures 1A and 1B), with a central parallel α-helical dimer of the phospho-Rab binding domain of RILPL2 bridging two molecules of pRab8a via hydrophobic and polar interactions (Figure 1C). As depicted by the domain organization of RILPL1/2 (Figure 1D), the topology of the complex on Golgi membranes would be consistent with the RH1 domain of RILPL2 (1–106) oriented above the complex in the orientation shown in Figure 1A to enable interactions with the GTD of MyoVa. Both molecules of pRab8a in the complex have GTP in the nucleotide pocket and their switch 1 and 2 conformations resemble the structure of active Rab8a (PDB: 4lhw; Guo et al., 2013). Each pRab8a molecule interacts with both α helices of the effector, burying approximately 625 Å^2 of surface area at each interface. The dual α-helical interactions are restricted to the N-terminal segment of RILPL2. As the coiled coil extends toward the C termini, a single α helix interacts with each Rab monomer by interfacing with switch 1 and strand β2 of the interswitch region (Figure 1E). The C termini of pRab8a (177–207) and RILPL2 (160–211) would reside proximal to the membrane, as indicated with dashed lines following helix α5 of

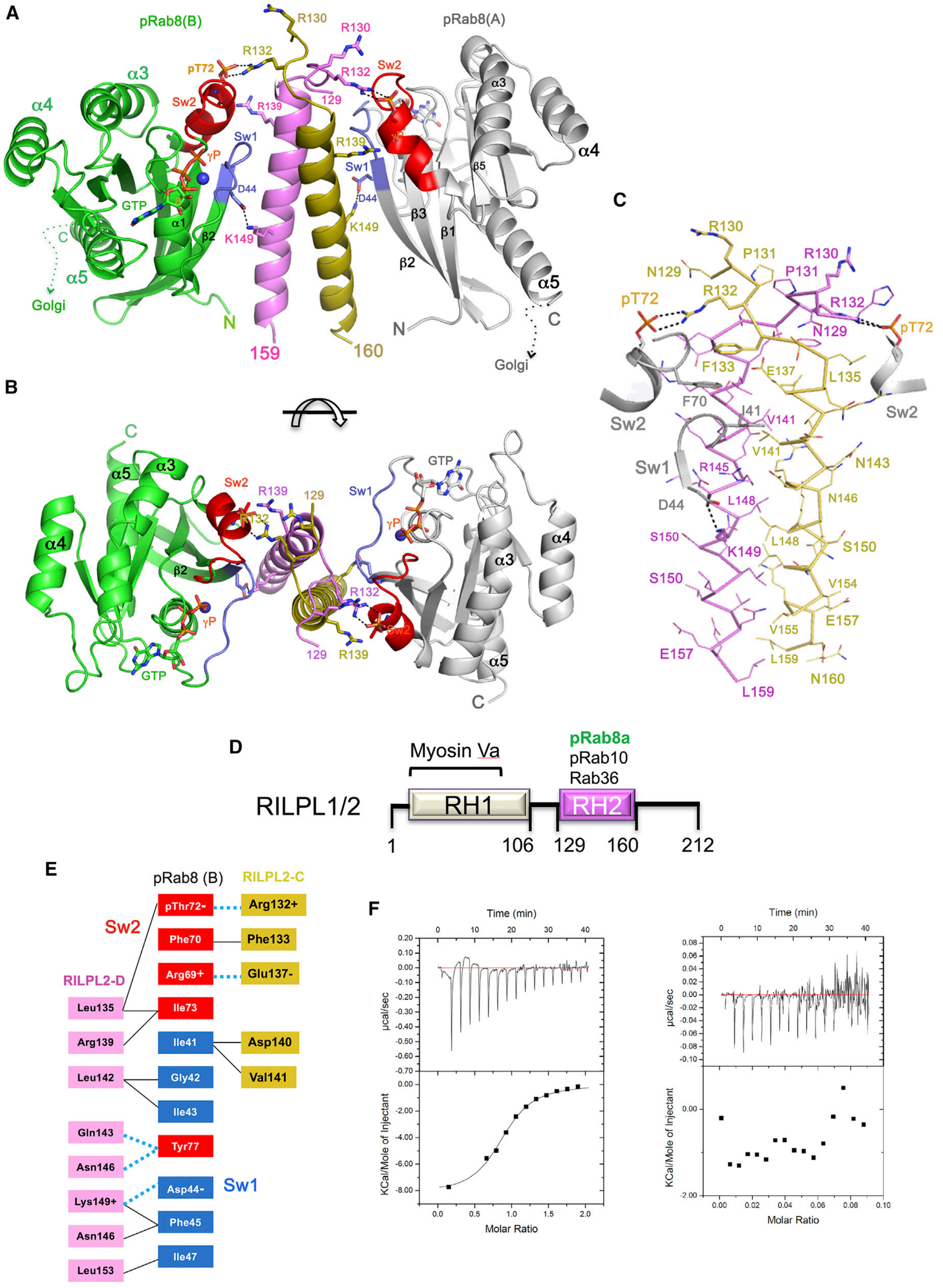

Figure 1. Structure of pRab8a in Complex with the Phospho-Rab Binding Domain of RILPL2

(A) Heterotetrameric assembly of two pRab8a molecules bridged by a central α-helical dimer of the phospho-Rab binding domain of RILPL2 (129–165). The two chains of RILPL2 are in magenta and dark yellow. For pRab8a, switch 1 is shown in blue, switch 2 in red.

(legend continued on next page)

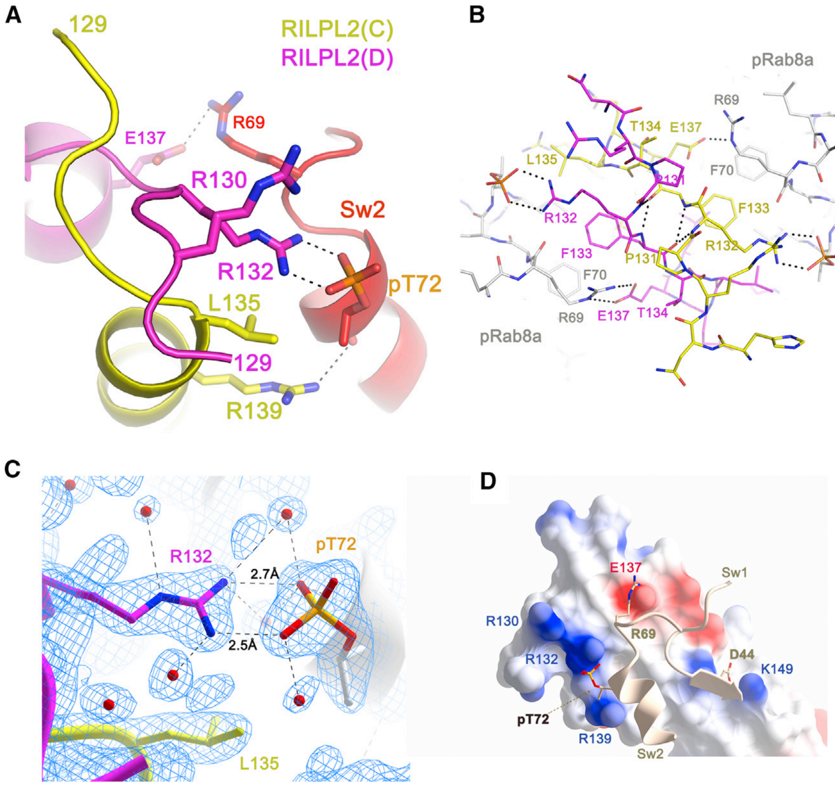

Figure 2. Structural Details of pT72 Recognition by the X-cap of RILPL2

(A) View of an interface between pRab8a and the dimer of RILPL2.

(B) Stick model of the interactions at the X-cap of RILPL2.

(C) Electron density ($2F_o - F_c$, 1.2σ) at the site of pT72 (chain A) binding to $R132^{RL2}$ (chain D, magenta). The side chain of $L135^{RL2}$ from chain C of RILPL2 lies within van der Waals contact (4 Å) of the β-branched methyl group of pT72.

(D) Electrostatic surface rendering of the X-cap. Blue is positive and red is negative, while switch 1 and 2 of pRab8a are ribbons with key residues represented as sticks.

pRab8a (Figure 1A). In the ensuing discussions, the acronyms "RL2" and "R8" will be used in superscript format to denote RILPL2 and Rab8a residues, respectively.

The affinity of the interaction between pRab8a and the phospho-Rab binding domain (129–165) of RILPL2 was evaluated by isothermal titration calorimetry (Figure 1F, Left). The experiment revealed a K_d = 3.3 μM ($\pm$0.5) for the interaction, which indicates a relatively weak affinity that is similar to other physiological Rab:effector complexes (Khan and Menetrey, 2013). RILPL2 is a preformed dimer in solution, as indicated by static light scattering coupled to gel filtration chromatography (Figure S2). There were no detectable interactions between non-phosphorylated Rab8a(GTP) and RILPL2 (Figure 1F, Right).

Phosphorylated Switch 2 of Rab8a Interacts with an "X-cap" Region of RILPL2

The N termini of the RILPL2 dimer ($N129^{RL2}$-$T134^{RL2}$) cross over in an extended conformation preceding the first α-helical turn, forming an X-shaped cap (X-cap) over the coiled coil (Figures 2A and 2B). The X-cap facilitates interactions of both monomers of RILPL2 with a single phosphorylated switch 2 of pRab8a. This X-cap contains two conserved arginines ($R130^{RL2}$ and $R132^{RL2}$) that previous mutagenesis analysis showed were required for interaction with pRab8a (Steger et al., 2017). Intimate contacts within the X-cap include reciprocal backbone hydrogen bonds between residues $R132^{RL2}$-$Phe133^{RL2}$ that resemble a short antiparallel β sheet (Figure 2B). The phosphate moiety from pT72 interacts with the guanidino group (NH1/NH2) of $R132^{RL2}$ on both sides of the symmetric complex, with O/N distances between 2.5 and 2.9 Å (Figure 2C). In contrast to these direct electrostatic and hydrogen-bonding interactions, the side chain of $R130^{RL2}$ is more distant from pT72 (>6 Å). An electrostatic surface map of RILPL2 reveals the strongly positive charges at the X-cap that enable recognition of pRab8a (Figure 2D). In addition to electrostatic contacts, the X-cap residue $F133^{RL2}$ contributes to a complementary hydrophobic interface with $F70^{R8}$ and $I73^{R8}$ from switch 2. The side chain of $T134^{RL2}$ acts as a capping residue by nucleating the α helix via a hydrogen bond (3.2 Å) to the backbone NH of $E137^{RL2}$. Therefore, the term X-cap is appropriate for this region of RILPL2.

Mutational Analyses of the Binding Interface

The contribution of RILPL2 residues to complex formation with pRab8a was evaluated by mutagenesis. LRRK2-phosphorylated Rab8a was subjected to co-immunoprecipitation studies in cells using wild-type and mutant forms of full-length RILPL2. For these studies a pathogenic LRRK2[R1441G] mutant was overexpressed to ensure maximal phosphorylation of Rab8a. We also treated cells with and without a potent and selective LRRK2 inhibitor termed MLi-2 (Fell et al., 2015) for 90 min to induce dephosphorylation of Rab8a and, therefore, block association with RILPL2 (Figures 3A and S3). These results revealed that

(B) View of the complex down the 2-fold axis of the heterotetramer, 90° relative to orientation in (A).

(C) Stick model of the RH2 domain of RILPL2. Rabs are stripped from the complex in this view, except for short segments of switch 1 and switch 2 (gray sticks).

(D) Domain organization of RILPL1/2 showing the RH domains and their interacting partners. The sequence corresponds to RILPL2.

(E) Simplified representation of the Rab:RILPL2 interface showing contacts between one molecule of Rab8a and the dimer of RILPL2. Polar interactions are indicated in dotted blue lines. Switch 1 (Sw1) and switch 2 are indicated.

(F) Isothermal titration calorimetry analyses of the interactions between pRab8a and the phospho-Rab binding domain of RILPL2. Left, titration of RILPL2 (residues 129–165) into pRab8a(GTP). Right, titration of RILPL2 into Rab8a(GTP).

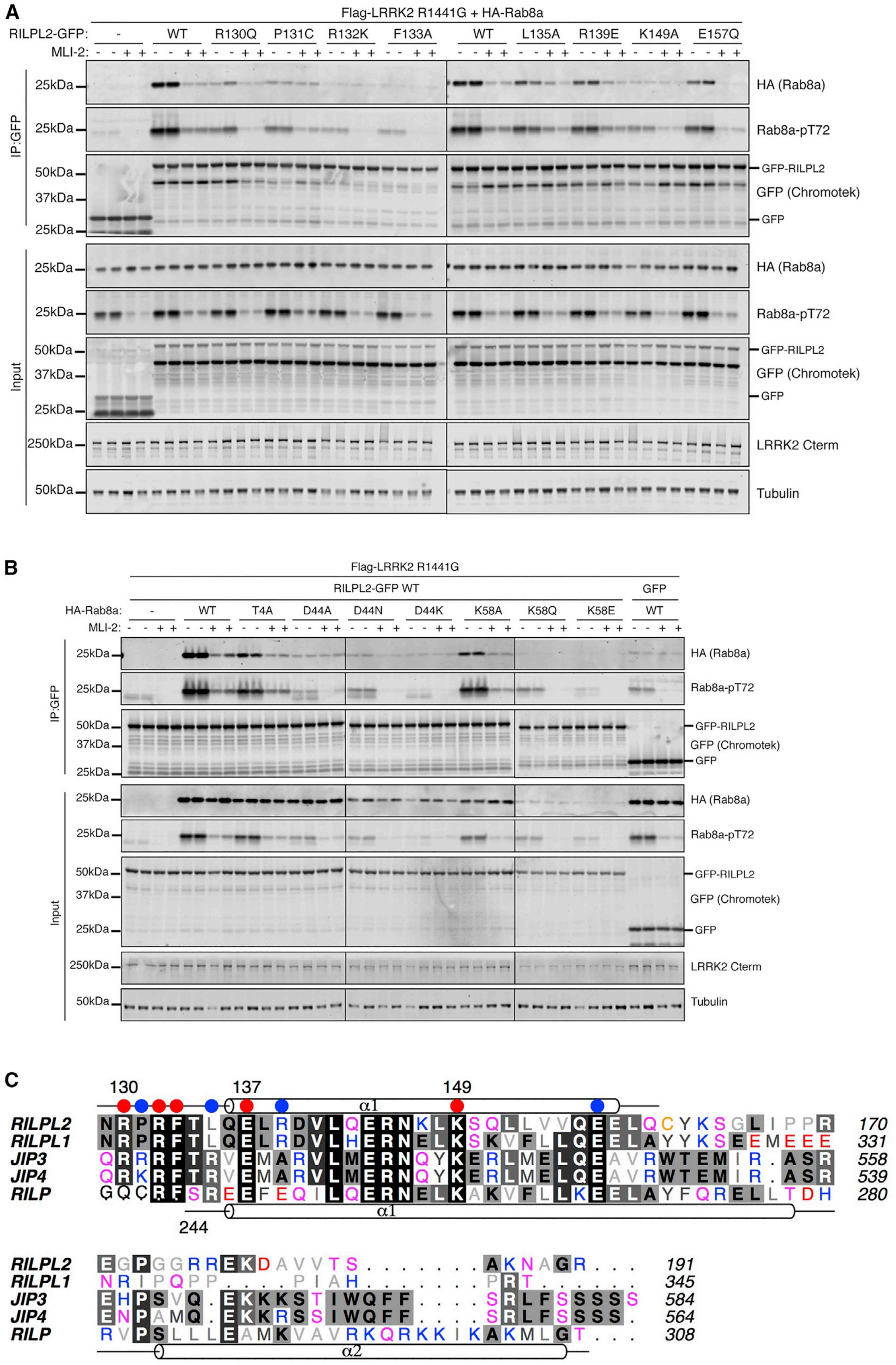

Figure 3. Mutational Analyses Reveal Hotspots of pRab8a:RILPL2 Interactions

(A) HEK293 cells were transiently transfected with constructs expressing Flag-LRRK2[R1441G], HA-Rab8a and WT or mutant RILPL2-GFP. At 48 h post transfection, cells were treated with ±500 nM MLi-2 for 90 min and then lysed. Upper panel, labeled IP:GFP: RILPL2-GFP was immunoprecipitated using GFP

(legend continued on next page)

all mutations of R132^{RL2}, which directly interacts with pT72, abolished the interaction with pRab8a in cells. The exquisite specificity of this contact is reflected by the R132K^{RL2} mutation, which was sufficient to abolish the interaction (Figure 3A). Mutation of K149^{RL2}—which forms a salt bridge with D44^{R8} (switch 1)—also abolishes the interaction between RILPL2 and pRab8a. This interaction, as well as hydrophobic packing of I41^{R8} against the RILPL2 α-helical dimer, presumably encodes GTP-dependent switch 1 specificity. Modest effects were observed with mutations of R139^{RL2} and L135^{RL2}, which form contacts with switch 2 adjacent to the R132^{RL2}:pT72 interaction. L135 packs against the β-branched methyl substituent of pT72, while R139 forms a hydrogen bond with the backbone carbonyl oxygen of pT72 (Figures 2A and 2C).

One apparent inconsistency between the structure and the mutagenesis experiments is the contribution of R130^{RL2} toward complex formation. Mutational studies reveal that R130Q^{RL2} (Figure 3A), as well as R130K^{RL2}, R130E^{RL2}, and R130A^{RL2} (Figure S3), severely compromise interactions with pRab8a, although to a lesser extent than R132^{RL2} mutants. As previously mentioned, the side chain of R130^{RL2} lies more than 6 Å away from pT72. There are two mechanisms by which the guanidino group of R130^{RL2} could contribute to complex formation. One is through long-range electrostatic interactions with pT72. A second mechanism may be an indirect stabilization of the key R132^{RL2}:pT72 contacts through stacking interactions with the side chain of R132^{RL2}. More detailed analyses of a full-length construct of RILPL2 and its affinity toward pRab8a is described below.

In addition to RILPL2, key residues in switch 1 and the N terminus of pRab8a were mutated to evaluate contributions to complex formation (Figure 3B). Most mutations in Rab8a tested likely disrupted protein stability, binding with other key interactors or phosphorylation by LRRK2 and, therefore, it was not possible to reliably evaluate the role of these residues in RILPL2 interaction *in vivo*. However, Rab8a mutations T4A^{R8} and K58A^{R8} were still phosphorylated by LRRK2 and were able to bind RILPL2, suggesting that these residues are not critical for this interaction.

The hotspots for pRab8a:RILPL2 interactions, as gleaned from cellular interactions with RILPL2 mutants, are shown with circles above the sequences of RILP family proteins (Figure 3C). The red circles denote essential residues for recognition of pRab8a(GTP) and are mostly conserved in the phospho-Rab binding domains. Arg139^{RL2} mutations have modest effects on Rab8a recognition (Figures 3A and S3), and are not conserved (blue circle). Pro131^{RL2} is variable in this family and the mutant Pro131A^{RL2} does not affect binding, suggesting that sequence variability is possible within the X-cap.

GTP Dependency of the Interaction between pRab8a and RILPL2

The GTP dependency of pRab8a interactions with full-length RILPL2 were investigated using *in vitro* pull-downs (Figure 4A). The interaction with RILPL2 *in vitro* is dependent on both the GTP conformation and phosphorylated T72 for Rab8a. Non-phosphorylated Rab8a and pRab8a(GDP) did not interact measurably with RILPL2.

To further investigate the GTP dependency in cells, we co-expressed RILPL2 with either wild-type Rab8a, Rab8a[Q67L] (GTP trapped conformation) or Rab8a[T22N] (GDP bound conformation) in the presence of pathogenic LRRK2[Y1699C] to induce maximal Rab8a phosphorylation (Figures 4B and S4). For these experiments, cells were treated ± LRRK2 MLi-2 inhibitor and phosphorylation of wild-type and mutant Rab8a was assessed in cell extracts as well as RILPL2 immunoprecipitates. Strikingly, this revealed that both cell lysates and immunoprecipitates of the pRab8a[Q67L] GTP locked mutant, MLi-2 failed to induce dephosphorylation of Rab8a over a 90-min period (Figure 4B). As mentioned previously, PPM1H has been identified as the phosphatase that dephosphorylates Rab GTPases (Berndsen et al., 2019). One interpretation of this observation is that the pRab8a[Q67L] GTP locked conformation remains stably associated with RILPL2 over this period and is thus protected from PPM1H. However, it cannot be excluded that PPM1H acts exclusively on pRab8a(GDP) in the cytosol, although *in vitro* studies demonstrated that PPM1H dephosphorylated pRab8a(GTP) and pRab8a(GDP) with similar efficiency (Berndsen et al., 2019). In comparison, MLi-2 treatment induced significant dephosphorylation of wild-type Rab8a that is presumably interconverting between the GTP and GDP conformation (Figure 4B). The GDP-locked conformation of Rab8a[T22N] was expressed at much lower levels in cell than wild-type or Rab8a[Q67L] (Figure S4). Nevertheless, no association of RILPL2 with Rab8a[T22N] was observed. Furthermore, phosphorylation of Rab8a[T22N] was not observed which is consistent with the GTP bound conformation being regulated by LRRK2.

Binding of pRab8a to Full-Length RILPL2 Is Enhanced by MyoVa

Relative to the isolated RH2 domain, all observed interactions *in vitro* between pRab8a and full-length RILPL2 were weak and unsuitable for isothermal titration calorimetry (e.g., Figure 4A). However, in the presence of the GTD of MyoVa, we observed a small but significant enhancement in affinity between pRab8a and RILPL2. Therefore, we utilized pull-down assays to characterize pRab8a:RILPL2 complex formation in the presence of MyoVa (Figures 5 and S5). The binary interactions between MyoVa and mouse RILPL2 have been measured previously

binder Sepharose and immunoprecipitates evaluated by immunoblotting with the indicated antibodies. Immunoblots were developed using the LI-COR Odyssey CLx western blot imaging system with the indicated antibodies at 0.5–1 μg/mL concentration. Lower panel, labeled input: 10 μg whole-cell lysate was subjected to LI-COR immunoblot analysis. Each lane represents cell extract obtained from a different dish of cells. Similar results were obtained in two separate experiments. (B) Same as A, but HEK293 cells were transiently transfected with WT or mutant HA-Rab8a as well as Flag-LRRK2[R1441G] and RILPL2-GFP WT. At 48 h post transfection, cells were treated with ±500 nM MLi-2 for 90 min and then lysed. RILPL2-GFP was immunoprecipitated using GFP binder Sepharose and as in (A), immunoprecipitates and input were evaluated by immunoblotting with the indicated antibodies. Each lane represents cell extract obtained from a different dish of cells. Similar results were obtained in two separate experiments.

(C) Sequence alignment of the first α helix (α1) of the RILP family RH2 domains. Residues corresponding to the second α helix (α2) of RILP are not shown. Red circles are hotspots for the interactions where mutations severely reduce affinity between pRab8a and RILPL2. Blue circles indicate residues that are tolerant to mutations. The α-helical secondary structure above the alignment corresponds to RILPL2.

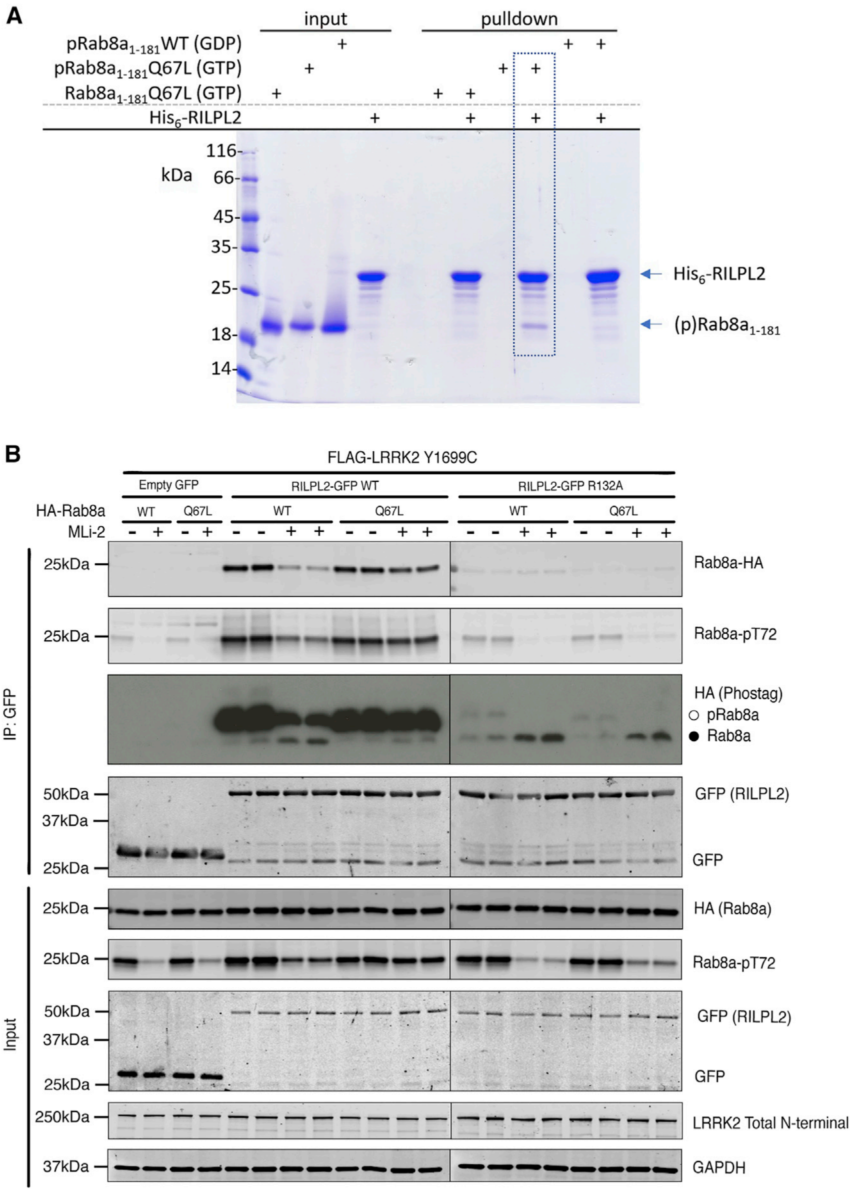

Figure 4. Evidence that RILPL2 Binds to the GTP Bound Conformation of Phosphorylated Rab8a in Cells

(A) Direct *in vitro* pull-downs were performed using purified His_6-tagged RILPL2 (full length) as bait and untagged Rab8a as prey. Rab8a species were either non-phosphorylated (Rab8a) or phosphorylated (pRab8a). The GTP forms were stabilized via the Q67L mutation in switch 2. The GDP form of Rab8a was prepared by *in vitro* exchange using wild-type (WT) Rab8a before the phosphorylation reaction to generate pRab8a(GDP). Protein concentrations were 10 μM for bait and prey, inputs are 2 μg; n ≥ 3, Coomassie stain for visualization. Dotted lines emphasize that only pRab8a(GTP) binds to RILPL2.

(B) HEK293 cells were transiently transfected with constructs expressing the indicated components. 24 h post transfection, cells were treated with ±100 nM MLi-2 for 90 min and then lysed. Upper panel, labeled IP:GFP: RILPL2-GFP was immunoprecipitated using GFP binder Sepharose and immunoprecipitates evaluated by immunoblotting with the indicated antibodies. Immunoblots were developed using the LI-COR Odyssey CLx western blot imaging system with the indicated antibodies at 0.5–1 μg/mL concentration. Lower panel, labeled input: 10 μg whole-cell lysate was subjected to LI-COR immunoblot analysis. Each lane represents cell extract obtained from a different dish of cells. Similar results were obtained in two separate experiments.

(K_d = 0.3 μM; Wei et al., 2013), and are 10-fold stronger than pRab8a interactions with the RH2 domain. In the presence of MyoVa, there is a clear increase in affinity between pRab8a and full-length RILPL2 (Figure 5A). This interaction is dependent on phosphorylated T72, since Rab8a(GTP) does not interact with the RILPL2:MyoVa complex. There are no observable interactions between pRab8a/Rab8a and the GTD of MyoVa (Figures 5B and S5).

The pull-downs were quantified to show the relative increase in binding of pRab8a:RILPL2 in the presence of MyoVa (Figure 5C). These data suggest that MyoVa(GTD) binding to the RH1 domain of RILPL2 enhances the affinity between the RH2 domain and pRab8a. A model of RILPL2 was built using the structure of the mouse RH1 domain (PDB: 4kp3) together with the human RH1 domain (Figure 5D). The side-by-side ribbon and electrostatic surface model shows that the two regions have complementary charges separated by a 20-residue flexible linker. In the absence of MyoVa, the RH1/RH2 domains of RILPL2 could interact in *cis*, thereby sterically blocking the X-cap and reducing the affinity of RH2 for pRab8a.

JIP3/4 Bind to LRRK2-Phosphorylated Rab10

Sequence comparisons suggest that two other scaffolding proteins previously implicated in JNK signaling, namely JIP3 (gene *MAPK8IP3*; Kelkar et al., 2000) and JIP4 (gene *SPAG9*; Kelkar et al., 2005), likely possess a phospho-Rab binding domain. These proteins have key residues equivalent to $R130^{RL2}$, $R132^{RL2}$, and $K149^{RL2}$ in their RH2 domain (Figure 3C). We, therefore, tested whether full-length JIP3 or JIP4 would bind to LRRK2-phosphorylated Rab8a or Rab10 in cells employing the co-expression assay utilized above to assess interaction of pRab8a with RILPL2 (Figures 6A and S6). These results revealed that both JIP3 and JIP4 specifically associated with LRRK2-phosphorylated Rab10. Addition of Mli-2 ablated interaction of JIP3 and JIP4 with Rab10 consistent with the interaction being phosphorylation dependent (Figure 6A). In contrast, a weak interaction just above background was observed between Rab8a and JIP3/4, which was not dependent upon phosphorylation by LRRK2 (Figure S6). This emphasizes that phospho-Rab binding domains are likely to display selectivity for different phosphorylated Rab proteins. Further work is required to identify

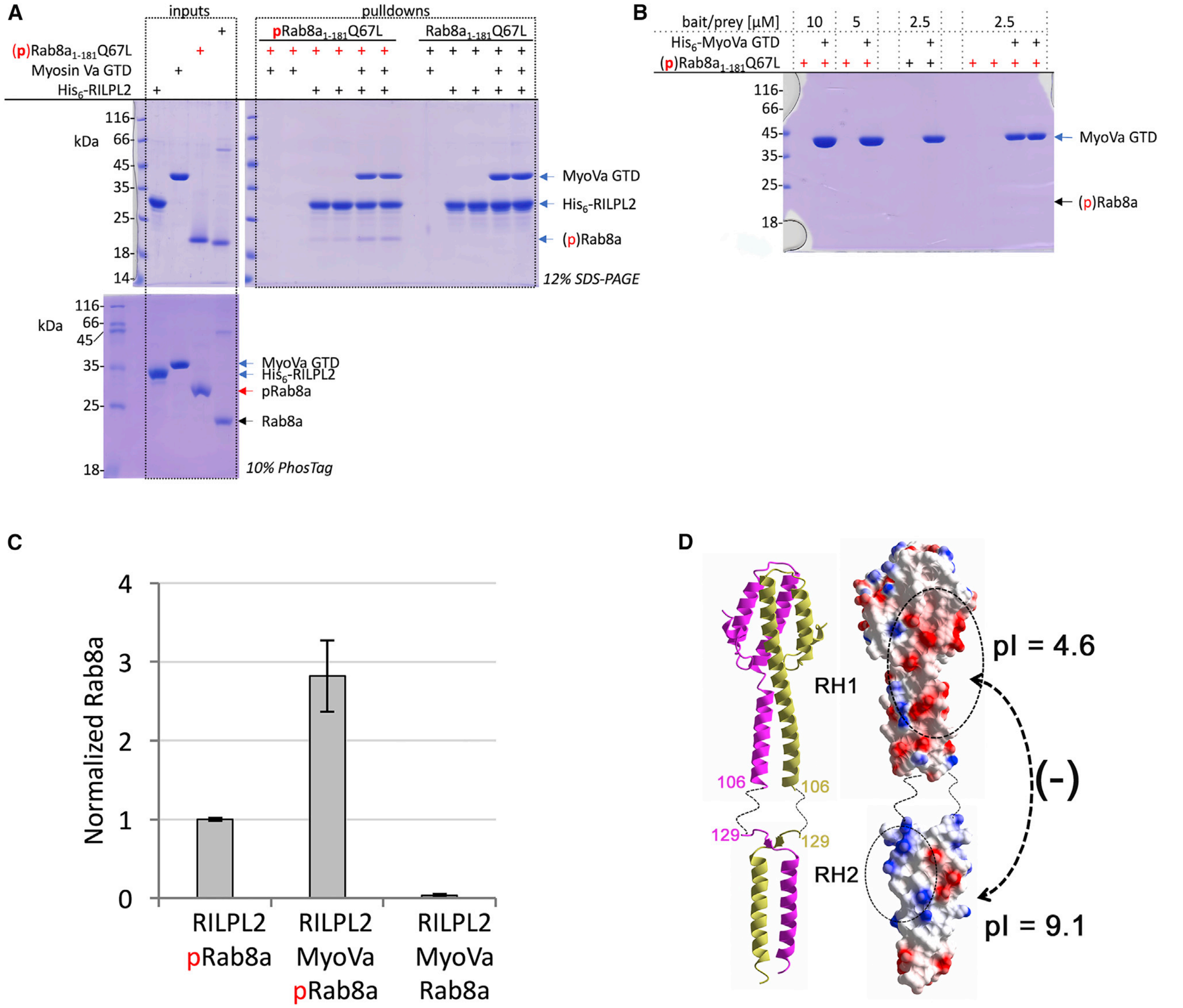

Figure 5. MyoVa Interactions with the RH1 Domain Enhance the Affinity of RILPL2 to pRab8a

(A) Pull-downs of (p)Rab8a and RILPL2 in the presence of MyoVa(GTD). Input proteins are in the upper left panel, while duplicate pull-downs are shown to the right. Phosphorylated Rab8a (pRab8a) is highlighted in the pull-down lanes with red (+) labels. Bait and prey proteins were used at 2.5 μM.

(B) Control experiment showing that no interactions are observed between His_6-tagged MyoVa and pRab8a/Rab8a.

(C) Quantification of densitometry readings of pRab8a pull-downs from three independent experiments ($p < 0.005$).

(D) Modeling of full-length RILPL2 using ribbons and electrostatic surfaces. The RH1 domain of mouse RILPL2 was connected to the RH2 domain of human RILPL2. Residue numbers correspond to the human protein.

which sets of phospho-Rab proteins interact with JIP3/4 and how these complexes regulate cytoskeletal dynamics through the microtubule network (Figure 6B).

DISCUSSION

The structure of the Rab binding domain of RILP bound to Rab7 has been determined previously (Figure 7A) (Wu et al., 2005). Similar to RILPL2, the effector forms a parallel coiled coil that binds two Rab7 molecules as a heterotetrameric complex. RILP does not have an X-cap at the N terminus—the first few residues in the structure beginning at Cys241 are disordered until the start of the core helix at Glu246 (Figures 7A and 7B). Following helix α1 of the Rab binding domain of RILP, a loop brings a second antiparallel helix α2 back toward the α1 dimer and is stabilized by hydrophobic interactions. Helix α2 is unlikely to be conserved in RILPL1/2 given a string of glycine and proline residues in these proteins following helix α1, which would be disruptive to secondary structures (Figure 3C). Interestingly, LRRK1 phosphorylation of Rab7 at Ser72 (switch 2) has recently been shown to promote interactions with RILP (Hanafusa et al., 2019). The RILP construct used for recombinant expression begins at C241 and the PDB file (PDB: 1yhn) for the complex RILP:Rab7 contains the coordinates for RILP beginning at

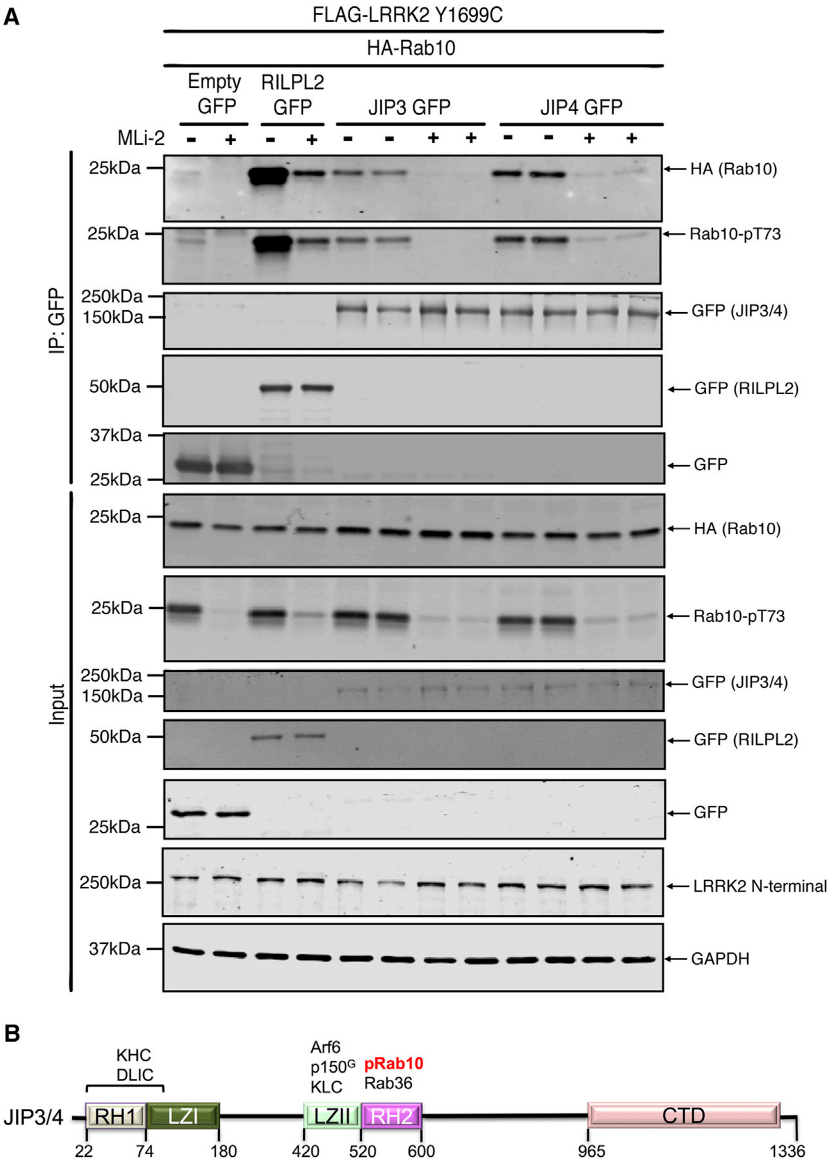

Figure 6. Evidence that JIP3 and JIP4 Bind to LRRK2-Phosphorylated Rab10 in Cells

(A) HEK293 cells were transiently transfected with constructs expressing the indicated components. At 24 h post transfection, cells were treated with ±100 nM MLi-2 for 90 min and then lysed. Upper panel, labeled IP:GFP: RILPL2-GFP, JIP3-GFP, JIP4-GFP were immunoprecipitated using GFP binder Sepharose and immunoprecipitates evaluated by immunoblotting with the indicated antibodies. Immunoblots were developed using the LI-COR Odyssey CLx western blot imaging system with the indicated antibodies at 0.5–1 μg/mL concentration. Lower panel, labeled input: 10 μg whole-cell lysate was subjected to LI-COR immunoblot analysis. Each lane represents cell extract obtained from a different dish of cells. Similar results were obtained in two separate experiments.

(B) Domain organization of JIP3 and JIP4. Sequence numbers correspond to JIP3, the interacting partners are shown above the cartoon, and $p150^{G}$ refers to $p150^{Glued}$. The figure is adapted from the recent structure of the RH1-LZI domain of JIP3 (Vilela et al., 2019). KHC, kinesin heavy chain; KLC, kinesin light chain; DLIC, dynein light intermediate chain.

S244 ($T134^{RL2}$). It is possible that an X-cap could form and bind to pSer72 (Rab7) given a modest extension of the RILP polypeptide toward the N terminus to stabilize backbone antiparallel hydrogen bonds. Although RILP lacks a positive residue (Q240), the structure of pRab8a:RILPL2 reveals that $Arg130^{RL2}$ is distant from pThr72 and may be tolerated as a glutamine within a possible X-cap in the RILP:pRab7 complex.

Unphosphorylated Rab8a is a promiscuous small GTPase and its structures in complex with effectors OCRL1 and MICAL have also been determined. The inositol-5-phosphatase OCRL1 is recruited to Rab8a membranes and regulates aspects of endosomal/Golgi trafficking (Mehta et al., 2014). Inherited X-linked mutations in OCRL1 lead to oculocerebrorenal syndrome of Lowe, which is associated with intellectual disability, cataracts, and renal dysfunction. Pull-downs using endogenous and hemagglutinin-tagged Rab8a reveal that OCRL1 does not bind to pRab8a (Steger et al., 2017). Superposition of the Rab8a:OCRL1 onto the pRaba:RILPL2 complex reveals a steric clash of non-hydrogen atoms between switch 2 and OCRL1 (Figures 7C and 7D). Switch 2 phosphorylation also disrupts interactions between Rab8a and GDI as well as Rabin8, a GEF for Rab8a (Steger et al., 2016). Along with GAPs, these Rab modulators interact with a broad segment of the switch 1/2 interface and would clash with pT72. In contrast to OCRL1, the effector MICAL can be docked onto pRab8a without steric problems (data not shown). Parallel coiled coils as Rab binding motifs are commonly exploited by effectors for membrane trafficking (Khan and Menetrey, 2013). However, the RILPL2-RH2 motif described here adopts a unique X-cap structure that resembles a short antiparallel β sheet at the N terminus of the coiled coil. The conformation enables positioning of Arg residues that recognize the phosphate on switch 2. The X-cap is likely a conserved feature among the RILP and JIP family of effector proteins.

A model for LRRK2 control of Rab trafficking is shown in Figure 8. Recent *in vitro* and cellular data are consistent with active Rab29(GTP) recruitment of the effector LRRK2 onto membranes where it phosphorylates substrates, such as Rab8a (Gomez et al., 2019; McGrath et al., 2019). The post-translational modification is situated at the edge of the switch/interswitch region of pRab8a and facilitates tuning of the strength of interacting proteins, including effectors, GAPs, GEFs, and GDI. The phosphospecific effector, RILPL2, is recruited onto membranes possibly as a preformed complex with MyoVa. Autosomal dominant variants of LRRK2 enhance the levels of pRab10 up to a maximum of 4% of total Rab10 in cells, which is a modest 2- to 4-fold increase over basal phosphorylation (Ito et al., 2016; Karayel et al., 2019). The effects arising from a small change in LRRK2-phosphorylated

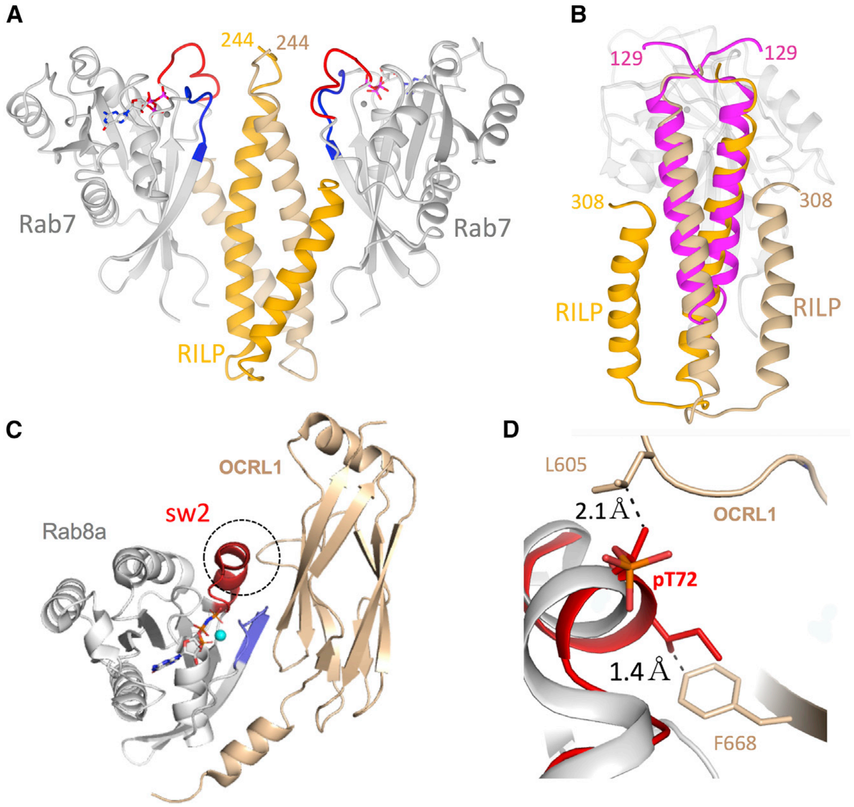

Figure 7. Structural Comparisons of Rab: Effector Complexes

(A) Structure of Rab7 in complex with the Rab binding domain of RILP.

(B) Superposition of RILPL2 onto a single binding interface of Rab7:RILP, showing conservation of the α-helical coiled coil. The figure is rotated 90° along the horizontal axis, relative to (A).

(C) Structure of Rab8a in complex with OCRL1. The dashed circle denotes the region that sterically clashes with pT72 of pRab8a.

(D) Close-up view of the switch 2 region denoted by the dashed circle. Here, pRab8a (red) from the complex with RILPL2 is superimposed onto the structure of Rab8a (gray) in complex with OCRL1. The distances between the methyl groups from the β-branched sidechains of pT72 and Ile71 are shown to highlight the steric clashes.

Rabs may be amplified through several mechanisms. One is the inability of Rabs to interact with GAPs, thereby prolonging the lifetime of pRabs on membranes. A second mechanism is recruitment of phospho-specific RILPL1/2 at the expense of other effectors, such as OCRL1. Here, we demonstrate that MyoVa enhances the affinity between pRab8a and RILPL2. In addition, a region of MyoVa encoded by exon D, ahead of the C-terminal GTD, also interacts directly with Rab8a and Rab10 (Lindsay et al., 2013). Finally, the ternary complex of pRab8a:RILPL2:MyoVa may be resistant to PPM1H-mediated hydrolysis of pT72. Together these factors could partly explain how LRRK2 directs pRab8a/10 toward RILPL1/2-associated ciliary trafficking pathways.

STAR★METHODS

Detailed methods are provided in the online version of this paper and include the following:

- KEY RESOURCES TABLE
- LEAD CONTACT AND MATERIALS AVAILABILITY
- METHOD DETAILS
  - Protein Expression and Purification
  - Rab8a Nucleotide Exchange
  - *In Vitro* Kinase Assays
  - Phosphorylation of Rab8a
  - Crystallization, Data Collection and Refinement
  - Structural Analyses and Superpositions
  - Pulldown Assays, Isothermal Titration Calorimetry, and Static Light Scattering
  - Plasmids for Cellular Assays
  - Antibody Reagents
  - Culture and Transfection of Cells
  - Co-Immunoprecipitation of Rab GTPases and RILPL2, JIP3 and JIP4
  - Gel Electrophoresis and Immunoblot Analysis
  - PhosTag Gel Electrophoresis and Immunoblot Analysis
- DATA AND CODE AVAILABILITY

SUPPLEMENTAL INFORMATION

Supplemental Information can be found online at https://doi.org/10.1016/j.str.2020.01.005.

ACKNOWLEDGMENTS

We thank the excellent technical support of the MRC-Protein Phosphorylation and Ubiquitylation Unit (PPU) Cloning Service (Melanie Whightman), DNA Sequencing Service (coordinated by Gary Hunter), the tissue culture team (coordinated by Edwin Allen), Reagents and Services antibody and protein purification teams (coordinated by Hilary McLauchlan and James Hastie). This work was supported by Science Foundation Ireland Principal Investigator Awards (grant no. 12/IA/1239 to A.R.K.), Michael J. Fox Foundation for Parkinson's research (grant no. 6986 to D.R.A.; the Medical Research Council, grant no. MC_UU_12016/2 to D.R.A.); the pharmaceutical companies supporting the Division of Signal Transduction Therapy Unit (Boehringer-Ingelheim, GlaxoSmithKline, and Merck KGaA, to D.R.A.). A.R.K. would like to thank the Division of Newborn Medicine and Program in Cellular and Molecular Medicine for generous research support. Data were collected at the Northeastern Collaborative Access Team beamlines, which are funded by the National Institute of General Medical Sciences from the NIH (P41 GM103403). The Pilatus 6M detector on 24-ID-C beam line is funded by an NIH-ORIP HEI grant (S10 RR029205). This research used resources of the Advanced Photon Source,

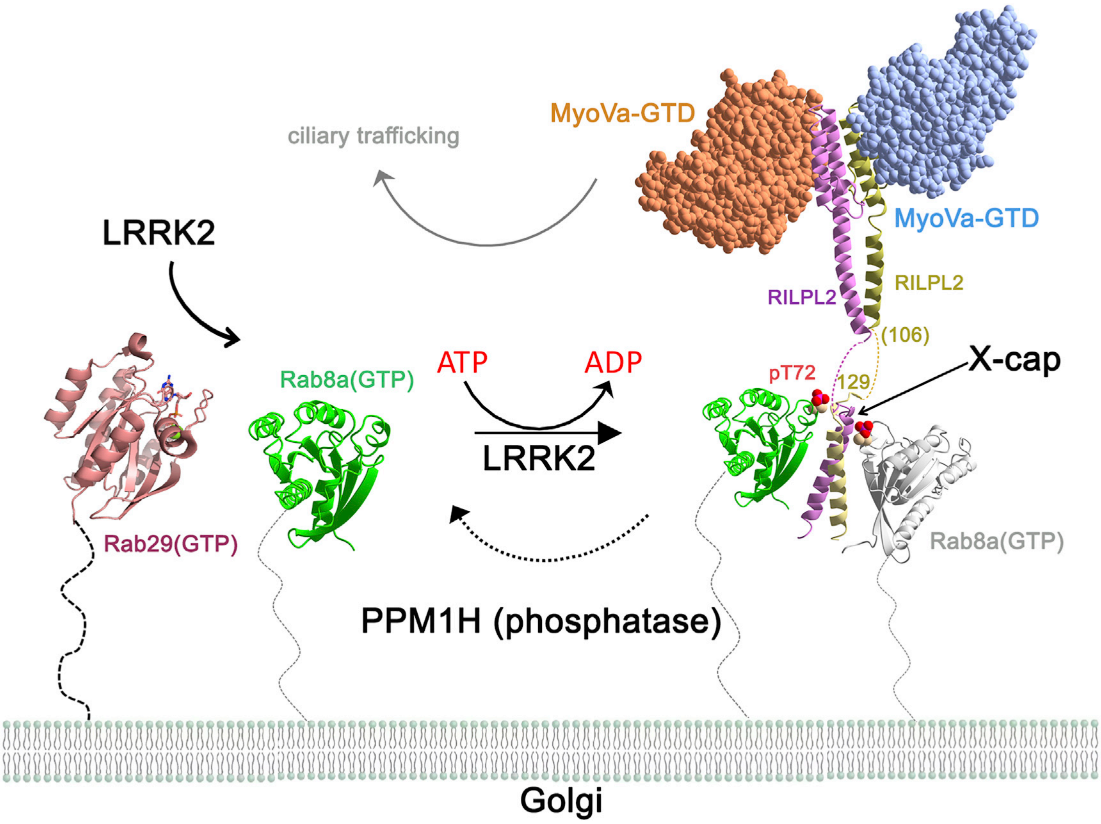

Figure 8. Model for the Control of Rab8a Functions by LRRK2

Rab29 recruits LRRK2 to membranes and Rab8a is subsequently phosphorylated by LRRK2. RILPL2 is then recruited to membranes by pRab8a via the X-cap. RILPL2 is an adaptor that links pRab8a to the GTD of MyoVa. The structure of the mouse complex of RILPL2 with myosin was used to generate this figure (PDB: 4kp3; Wei et al., 2013).

a U.S. Department of Energy (DOE) Office of Science User Facility operated for the DOE Office of Science by Argonne National Laboratory under contract no. DE-AC02-06CH11357.

AUTHOR CONTRIBUTIONS

D.W., E.P., P.P., D.R.A., and A.R.K. designed the experiments. D.W., E.P., P.P., E.McG., and A.R.K. performed the experiments. D.W., E.P., P.P., D.R.A., and A.R.K. wrote the manuscript.

DECLARATION OF INTERESTS

The authors declare no competing interests.

Received: August 31, 2019
Revised: November 27, 2019
Accepted: January 13, 2020
Published: February 3, 2020

REFERENCES

Adams, P.D., Afonine, P.V., Bunkoczi, G., Chen, V.B., Davis, I.W., Echols, N., Headd, J.J., Hung, L.W., Kapral, G.J., Grosse-Kunstleve, R.W., et al. (2010). PHENIX: a comprehensive Python-based system for macromolecular structure solution. Acta Crystallogr. D Biol. Crystallogr. *66*, 213–221.

Bardien, S., Lesage, S., Brice, A., and Carr, J. (2011). Genetic characteristics of leucine-rich repeat kinase 2 (LRRK2) associated Parkinson's disease. Parkinsonism Relat. Disord. *17*, 501–508.

Berndsen, K., Lis, P., Yeshaw, W.M., Wawro, P.S., Nirujogi, R.S., Wightman, M., Macartney, T., Dorward, M., Knebel, A., Tonelli, F., et al. (2019). PPM1H phosphatase counteracts LRRK2 signaling by selectively dephosphorylating Rab proteins. Elife *8*, https://doi.org/10.7554/eLife.50416.001.

Cantalupo, G., Alifano, P., Roberti, V., Bruni, C.B., and Bucci, C. (2001). Rab-interacting lysosomal protein (RILP): the Rab7 effector required for transport to lysosomes. EMBO J. *20*, 683–693.

Dhekne, H.S., Yanatori, I., Gomez, R.C., Tonelli, F., Diez, F., Schule, B., Steger, M., Alessi, D.R., and Pfeffer, S.R. (2018). A pathway for Parkinson's disease LRRK2 kinase to block primary cilia and Sonic hedgehog signaling in the brain. Elife *7*, e40202.

Dzamko, N., Inesta-Vaquera, F., Zhang, J., Xie, C., Cai, H., Arthur, S., Tan, L., Choi, H., Gray, N., Cohen, P., et al. (2012). The IkappaB kinase family phosphorylates the Parkinson's disease kinase LRRK2 at Ser935 and Ser910 during Toll-like receptor signaling. PLoS One *7*, e39132.

Emsley, P., Lohkamp, B., Scott, W.G., and Cowtan, K. (2010). Features and development of coot. Acta Crystallogr. D Biol. Crystallogr. *66*, 486–501.

Evans, P.R., and Murshudov, G.N. (2013). How good are my data and what is the resolution? Acta Cryst. *D69*, 1204–1214.

Fahn, S. (2003). Description of Parkinson's disease as a clinical syndrome. Ann. N. Y Acad. Sci. *991*, 1–14.

Fell, M.J., Mirescu, C., Basu, K., Cheewatrakoolpong, B., DeMong, D.E., Ellis, J.M., Hyde, L.A., Lin, Y., Markgraf, C.G., Mei, H., et al. (2015). MLi-2, a potent, selective, and centrally active compound for exploring the therapeutic potential and safety of LRRK2 kinase inhibition. J. Pharmacol. Exp. Ther. *355*, 397–409.

Fujimoto, T., Kuwahara, T., Eguchi, T., Sakurai, M., Komori, T., and Iwatsubo, T. (2018). Parkinson's disease-associated mutant LRRK2 phosphorylates

Rab7L1 and modifies trans-Golgi morphology. Biochem. Biophys. Res. Commun. *495*, 1708–1715.

Gomez, R.C., Wawro, P., Lis, P., Alessi, D.R., and Pfeffer, S.R. (2019). Membrane association but not identity is required for LRRK2 activation and phosphorylation of Rab GTPases. J. Cell Biol. *18*, 4157–4170.

Greggio, E., Zambrano, I., Kaganovich, A., Beilina, A., Taymans, J.M., Daniels, V., Lewis, P., Jain, S., Ding, J., Syed, A., et al. (2008). The Parkinson disease-associated leucine-rich repeat kinase 2 (LRRK2) is a dimer that undergoes intramolecular autophosphorylation. J. Biol. Chem. *283*, 16906–16914.

Guo, Z., Hou, X., Goody, R.S., and Itzen, A. (2013). Intermediates in the guanine nucleotide exchange reaction of Rab8 protein catalyzed by guanine nucleotide exchange factors Rabin8 and GRAB. J. Biol. Chem. *288*, 32466–32474.

Hanafusa, H., Yagi, T., Ikeda, H., Hisamoto, N., Nishioka, T., Kaibuchi, K., Shirakabe, K., and Matsumoto, K. (2019). LRRK1 phosphorylation of Rab7 at S72 links trafficking of EGFR-containing endosomes to its effector RILP. J. Cell Sci. *132*, https://doi.org/10.1242/jcs.228809.

Hou, X., Hagemann, N., Schoebel, S., Blankenfeldt, W., Goody, R.S., Erdmann, K.S., and Itzen, A. (2011). A structural basis for Lowe syndrome caused by mutations in the Rab-binding domain of OCRL1. EMBO J. *30*, 1659–1670.

Hui, K.Y., Fernandez-Hernandez, H., Hu, J., Schaffner, A., Pankratz, N., Hsu, N.Y., Chuang, L.S., Carmi, S., Villaverde, N., Li, X., et al. (2018). Functional variants in the LRRK2 gene confer shared effects on risk for Crohn's disease and Parkinson's disease. Sci. Transl. Med. *10*, eaai7795.

Hutagalung, A.H., and Novick, P.J. (2011). Role of Rab GTPases in membrane traffic and cell physiology. Physiol. Rev. *91*, 119–149.

Ito, G., Katsemonova, K., Tonelli, F., Lis, P., Baptista, M.A., Shpiro, N., Duddy, G., Wilson, S., Ho, P.W., Ho, S.L., et al. (2016). Phos-tag analysis of Rab10 phosphorylation by LRRK2: a powerful assay for assessing kinase function and inhibitors. Biochem. J. *473*, 2671–2685.

Jordens, I., Fernandez-Borja, M., Marsman, M., Dusseljee, S., Janssen, L., Calafat, J., Janssen, H., Wubbolts, R., and Neefjes, J. (2001). The Rab7 effector protein RILP controls lysosomal transport by inducing the recruitment of dynein-dynactin motors. Curr. Biol. *11*, 1680–1685.

Kabsch, W. (2010). Integration, scaling, space-group assignment and post-refinement. Acta Crystallogr. D. Biol. Crystallogr. *66*, 133–144.

Karayel, Ö., Tonelli, F., Winter, S.V., Geyer, P.E., Fan, Y., Sammler, E.M., Alessi, D.R., Steger, M., and Mann, M. (2019). Accurate MS-based Rab10 phosphorylation stoichiometry determination as readout for LRRK2 activity in Parkinson's disease. bioRxiv. https://doi.org/10.1101/819607.

Kelkar, N., Gupta, S., Dickens, M., and Davis, R.J. (2000). Interaction of a mitogen-activated protein kinase signaling module with the neuronal protein JIP3. Mol. Cell Biol. *20*, 1030–1043.

Kelkar, N., Standen, C.L., and Davis, R.J. (2005). Role of the JIP4 scaffold protein in the regulation of mitogen-activated protein kinase signaling pathways. Mol. Cell Biol. *25*, 2733–2743.

Khan, A.R., and Menetrey, J. (2013). Structural biology of Arf and Rab GTPases' effector recruitment and specificity. Structure *21*, 1284–1297.

Krissinel, E., and Henrick, K. (2004). Secondary-structure matching (SSM), a new tool for fast protein structure alignment in three dimensions. Acta Crystallogr. D Biol. Crystallogr. *60*, 2256–2268.

Liebschner, D., Afonine, P.V., Baker, M.L., Bunkóczi, G., Chen, V.B., Croll, T.I., Hintze, B., Hung, L.-W., Jain, S., McCoy, A.J., et al. (2019). Macromolecular structure determination using X-rays, neutrons and electrons: recent developments in Phenix. Acta Crystallogr. D Struct. Biol. *75*, 861–877.

Lindsay, A.J., Jollivet, F., Horgan, C.P., Khan, A.R., Raposo, G., McCaffrey, M.W., and Goud, B. (2013). Identification and characterization of multiple novel Rab-myosin Va interactions. Mol. Biol. Cell *24*, 3420–3434.

Lise, M.F., Srivastava, D.P., Arstikaitis, P., Lett, R.L., Sheta, R., Viswanathan, V., Penzes, P., O'Connor, T.P., and El-Husseini, A. (2009). Myosin-Va-interacting protein, RILPL2, controls cell shape and neuronal morphogenesis via Rac signaling. J. Cell Sci. *122*, 3810–3821.

Liu, Z., Bryant, N., Kumaran, R., Beilina, A., Abeliovich, A., Cookson, M.R., and West, A.B. (2018). LRRK2 phosphorylates membrane-bound Rabs and is activated by GTP-bound Rab7L1 to promote recruitment to the trans-Golgi network. Hum. Mol. Genet. *27*, 385–395.

McCoy, A.J., Grosse-Kunstleve, R.W., Adams, P.D., Winn, M.D., Storoni, L.C., and Read, R.J. (2007). Phaser crystallographic software. J. Appl. Crystallogr. *40*, 658–674.

McGrath, E., Waschbusch, D., Baker, B.M., and Khan, A.R. (2019). LRRK2 binds to the Rab32 subfamily in a GTP-dependent manner via its armadillo domain. Small GTPases, 1–14.

Mehta, Z.B., Pietka, G., and Lowe, M. (2014). The cellular and physiological functions of the Lowe syndrome protein OCRL1. Traffic *15*, 471–487.

Paisan-Ruiz, C., Jain, S., Evans, E.W., Gilks, W.P., Simon, J., van der Brug, M., Lopez de Munain, A., Aparicio, S., Gil, A.M., Khan, N., et al. (2004). Cloning of the gene containing mutations that cause PARK8-linked Parkinson's disease. Neuron *44*, 595–600.

Purlyte, E., Dhekne, H.S., Sarhan, A.R., Gomez, R., Lis, P., Wightman, M., Martinez, T.N., Tonelli, F., Pfeffer, S.R., and Alessi, D.R. (2017). Rab29 activation of the Parkinson's disease-associated LRRK2 kinase. EMBO J. *4*, 1–18.

Roosen, D.A., and Cookson, M.R. (2016). LRRK2 at the interface of autophagosomes, endosomes and lysosomes. Mol. Neurodegener. *11*, 73.

Schaub, J.R., and Stearns, T. (2013). The Rilp-like proteins Rilpl1 and Rilpl2 regulate ciliary membrane content. Mol. Biol. Cell *24*, 453–464.

Schindelin, J., Arganda-Carreras, I., Frise, E., Kaynig, V., Longair, M., Pietzsch, T., Preibisch, S., Rueden, C., Saalfeld, S., Schmid, B., et al. (2012). Fiji: an open-source platform for biological-image analysis. Nat. Methods *9*, 676–682.

Schulte, C., and Gasser, T. (2011). Genetic basis of Parkinson's disease: inheritance, penetrance, and expression. Appl. Clin. Genet. *4*, 67–80.

Steger, M., Diez, F., Dhekne, H.S., Lis, P., Nirujogi, R.S., Karayel, O., Tonelli, F., Martinez, T.N., Lorentzen, E., Pfeffer, S.R., et al. (2017). Systematic proteomic analysis of LRRK2-mediated Rab GTPase phosphorylation establishes a connection to ciliogenesis. Elife *6*, e31012.

Steger, M., Tonelli, F., Ito, G., Davies, P., Trost, M., Vetter, M., Wachter, S., Lorentzen, E., Duddy, G., Wilson, S., et al. (2016). Phosphoproteomics reveals that Parkinson's disease kinase LRRK2 regulates a subset of Rab GTPases. Elife *5*, e12813.

Vieweg, S., Mulholland, K., Bräuning, B., Kachariya, N., Lai, Y.-C., Toth, R., Sattler, M., Groll, M., Itzen, A., and Muqit, M.M.K. (2019). PINK1-dependent phosphorylation of Serine111 within the SF3 motif of Rab GTPases impairs effector interactions and LRRK2 mediated phosphorylation at threonine72. bioRxiv. https://doi.org/10.1101/764019.

Vilela, F., Velours, C., Chenon, M., Aumont-Nicaise, M., Campanacci, V., Thureau, A., Pylypenko, O., Andreani, J., Llinas, P., and Menetrey, J. (2019). Structural characterization of the RH1-LZI tandem of JIP3/4 highlights RH1 domains as a cytoskeletal motor-binding motif. Sci. Rep. *9*, 16036.

Waschbusch, D., Michels, H., Strassheim, S., Ossendorf, E., Kessler, D., Gloeckner, C.J., and Barnekow, A. (2014). LRRK2 transport is regulated by its novel interacting partner Rab32. PLoS One *9*, e111632.

Wei, Z., Liu, X., Yu, C., and Zhang, M. (2013). Structural basis of cargo recognitions for class V myosins. Proc. Natl. Acad. Sci. U S A *110*, 11314–11319.

West, A.B., and Cookson, M.R. (2016). Identification of bona-fide LRRK2 kinase substrates. Mov. Disord. *31*, 1140–1141.

Williams, C.J., Headd, J.J., Moriarty, N.W., Prisant, M.G., Videau, L.L., Deis, L.N., Verma, V., Keedy, D.A., Hintze, B.J., Chen, V.B., et al. (2018). MolProbity: more and better reference data for improved all-atom structure validation. Protein Sci. *27*, 293–315.

Winn, M.D., Ballard, C.C., Cowtan, K.D., Dodson, E.J., Emsley, P., Evans, P.R., Keegan, R.M., Krissinel, E.B., Leslie, A.G., McCoy, A., et al. (2011). Overview of the CCP4 suite and current developments. Acta Crystallogr. D Biol. Crystallogr. *67*, 235–242.

Wu, M., Wang, T., Loh, E., Hong, W., and Song, H. (2005). Structural basis for recruitment of RILP by small GTPase Rab7. EMBO J. *24*, 1491–1501.

Zimprich, A., Biskup, S., Leitner, P., Lichtner, P., Farrer, M., Lincoln, S., Kachergus, J., Hulihan, M., Uitti, R.J., Calne, D.B., et al. (2004). Mutations in LRRK2 cause autosomal-dominant parkinsonism with pleomorphic pathology. Neuron *44*, 601–607.

STAR★METHODS

KEY RESOURCES TABLE

REAGENT or RESOURCE	SOURCE	IDENTIFIER
Antibodies		
Rabbit anti LRRK2 N-term	University of Dundee	UDD3
Rabbit anti GFP	Chromotek	PABG1
Rabbit anti GFP	Cell Signaling Technology	#2956
Rat anti HA	Merck	3F10
Rabbit anti-pT72-Rab8a	Abcam	MJF-R20; ab231706
Mouse anti LRRK2 C-term	NeuroMAB	N241A/34; AB_10675136
Mouse anti Tubulin	Cell Signaling Technology	3873S
Mouse anti GAPDH	Santa Cruz Biotechnology	sc-32233
Goat anti-Rabbit IRDye 800CW	Licor	925-32211
Goat anti-Mouse IRDye 800CW	Licor	926-32210
Goat anti-Rat IRDye 680LT	Licor	925-68029
Goat anti-mouse IRDye 680LT	Licor	926-68020
Goat anti-Rat IgG HRP conjugated	Thermo Fisher Scientific	#31470
Bacterial and Viral Strains		
E. coli BL21 (DE3)	New England Biolabs	C2527
E. coli DH5α	New England Biolabs	C2987
Chemicals, Peptides, and Recombinant Proteins		
His_6-RILPL2 residue 129-165	Genscript	N/A
GDP	Sigma Aldrich	G7127
ATP	Sigma Aldrich	A2383
MIL-2 LRRK2 inhnibitor	MRC PPU Reagents Services	N/A
GST-MST3	MRC PPU Reagents Services	DU30889
Polyethylenimine PEI	Polysciences	23966
αGFP binder sepharose	MRC PPU Reagents Services	N/A
PhosTag reagent	MRC PPU Reagents Services	N/A
Ni-agarose fast flow	GE Healthcare	17531802
Critical Commercial Assays		
JCSG-plus crystallography screen	Molecular Dimensions	MD1-37
PACT premier crystallography screen	Molecular Dimensions	MD1-29
Isolate II Miniprep Kit	Bioline	BIO-52057
Deposited Data		
pRab8a:RILPL2 complex	This study	PDB: 6RIR
Rab7:RILP complex	Wu et al., 2005	PDB: 1YHN
Strcture of Rab8a:OCRL1 complex	Hou et al., 2011	PDB: 3QBT
MyosinVa GTD:RILPL2 complex	Wei et al., 2013	PDB: 4KP3
Experimental Models: Cell Lines		
HEK293	ATCC	CRL-1573
Oligonucleotides		
Rab8a mutagenesis primer for GG GAT ACC GCG GGT CAG GAA CGT TTT CGT AC	This study	N/A
Rab8a mutagenesis primer rev GT ACG AAA ACG TTC CTG ACC CGC GGT ATC CC	This study	N/A

(*Continued on next page*)

Continued

REAGENT or RESOURCE	SOURCE	IDENTIFIER
Recombinant DNA		
pET28a(+)-Rab8a$_{1-181}$Q67L	Genscript	N/A
pET28a(+)-Rab8a$_{1-181}$WT	This study	N/A
pET15b-RILPL2	Genscript	N/A
pET28a(+)-MyosinVa GTD	Genscript	N/A
pCVM5 HA-empty	MRC PPU Reagents Services	DU49303
pcDNA5 GFP-empty	MRC PPU Reagents Services	DU13156
pCMV Flag-LRRK2 R1441G	MRC PPU Reagents Services	DU13077
pCMV Flag-LRRK2 Y1699C	MRC PPU Reagents Services	DU1316
pCMV HA-Rab8a WT	MRC PPU Reagents Services	DU35414
pCMV HA-Rab10 WT	MRC PPU Reagents Services	DU44250
pcDNA5D FRT/TO RILPL2-GFP WT	MRC PPU Reagents Services	DU27481
pcDNA5D FRT/TO RILPL2-GFP R130K	MRC PPU Reagents Services	DU68258
pcDNA5D FRT/TO RILPL2-GFP R130A	MRC PPU Reagents Services	DU68022
pcDNA5D FRT/TO RILPL2-GFP R130Q	MRC PPU Reagents Services	DU27521
pcDNA5D FRT/TO RILPL2-GFP R130E	MRC PPU Reagents Services	DU27520
pcDNA5D FRT/TO RILPL2-GFP P131A	MRC PPU Reagents Services	DU68030
pcDNA5D FRT/TO RILPL2-GFP P131C	MRC PPU Reagents Services	DU68031
pcDNA5D FRT/TO RILPL2-GFP P131K	MRC PPU Reagents Services	DU68256
pcDNA5D FRT/TO RILPL2-GFP P131R	MRC PPU Reagents Services	DU68257
pcDNA5D FRT/TO RILPL2-GFP R132K	MRC PPU Reagents Services	DU68023
pcDNA5D FRT/TO RILPL2-GFP R132A	MRC PPU Reagents Services	DU67110
pcDNA5D FRT/TO RILPL2-GFP R132Q	MRC PPU Reagents Services	DU68037
pcDNA5D FRT/TO RILPL2-GFP R132E	MRC PPU Reagents Services	DU27522
pcDNA5D FRT/TO RILPL2-GFP F133A	MRC PPU Reagents Services	DU68033
pcDNA5D FRT/TO RILPL2-GFP L135A	MRC PPU Reagents Services	DU68032
pcDNA5D FRT/TO RILPL2-GFP R139A	MRC PPU Reagents Services	DU68025
pcDNA5D FRT/TO RILPL2-GFP R139Q	MRC PPU Reagents Services	DU68024
pcDNA5D FRT/TO RILPL2-GFP R139E	MRC PPU Reagents Services	DU68026
pcDNA5D FRT/TO RILPL2-GFP K149A	MRC PPU Reagents Services	DU68029
pcDNA5D FRT/TO RILPL2-GFP K149Q	MRC PPU Reagents Services	DU68027
pcDNA5D FRT/TO RILPL2-GFP K149E	MRC PPU Reagents Services	DU68028
pcDNA5D FRT/TO RILPL2-GFP E157A	MRC PPU Reagents Services	DU68036
pcDNA5D FRT/TO RILPL2-GFP E157Q	MRC PPU Reagents Services	DU68034
pcDNA5D FRT/TO RILPL2-GFP E157K	MRC PPU Reagents Services	DU68035
pCMV HA-Rab8a Q67L	MRC PPU Reagents Services	DU39393
pCMV HA-Rab8a T22N	MRC PPU Reagents Services	DU39392
pCMV5 HA-Rab8a T4A	MRC PPU Reagents Services	DU68045
pCMV5 HA-Rab8a D44A	MRC PPU Reagents Services	DU68041
pCMV5 HA-Rab8a D44N	MRC PPU Reagents Services	DU68039
pCMV5 HA-Rab8a D44K	MRC PPU Reagents Services	DU68040
pCMV5 HA-Rab8a K58A	MRC PPU Reagents Services	DU68044
pCMV5 HA-Rab8a K58Q	MRC PPU Reagents Services	DU68042
pCMV5 HA-Rab8a K58E	MRC PPU Reagents Services	DU68043
pcDNA5D FRT/TO JIP3-GFP	MRC PPU Reagents Services	DU27721
pcDNA5D FRT/TO JIP4-GFP	MRC PPU Reagents Services	DU27684
pcDNA5D FRT/TO RILPL1-GFP	MRC PPU Reagents Services	DU27305
pCVM5 HA-empty	MRC PPU Reagents Services	DU49303

(*Continued on next page*)

Continued		
REAGENT or RESOURCE	SOURCE	IDENTIFIER
Software and Algorithms		
Phenix	Liebschner et al., 2019	https://www.phenix-online.org/
Coot	Emsley et al., 2010	https://www2.mrc-lmb.cam.ac.uk/personal/pemsley/coot/
CCP4i	Krissinel and Henrick, 2004; Winn et al., 2011	https://www.ccp4.ac.uk/ccp4i_main.php
PyMol	Schrödinger, LLC	https://pymol.org
ImageJ (Fiji)	Schindelin et al., 2012	https://imagej.nih.gov/ij/
XDS	Kabsch, (2010)	http://xds.mpimf-heidelberg.mpg.de/
Aimless	Evans and Murshudov, 2013	http://www.ccp4.ac.uk/html/aimless.html
Phaser	McCoy et al., 2007	http://www.ccp4.ac.uk/html/phaser.html
Other		
Superdex 75 (16/60)	GE Healthcare	28-9893-33
Superdex 200 (16/60)	GE Healthcare	28-9893-35
Superdex 75 (10/300)	GE Healthcare	17-5174-01
Superdex 200 (10/300)	GE Healthcare	17-5175-01
MonoS 5/50 GL	GE Healthcare	17-5168-01
PD10 column	GE Healthcare	17-0851-01
ITC-200 instrument	Malvern Panalytical	N/A
miniDAWN system SLS instrument	Wyatt Corp	N/A
Optilab rEX refractometer	Wyatt Corp	N/A
ZORBAX 300SB-C18 HPLC column	Agilent	N/A

LEAD CONTACT AND MATERIALS AVAILABILITY

Further information and requests for resources and reagents should be directed to and will be fulfilled by Lead Contact, Amir Khan (Amir.Khan@tcd.ie). This study did not generate new unique agents.

METHOD DETAILS

Protein Expression and Purification

The cDNA for Rab8a (residues 1-181, Q67L) lacking the flexible C-terminal tail was ordered from Genscript in a codon-optimized form to enable *E.coli* expression. The cDNA was cloned into pET28a at the NdeI/BamH1 sites for both of these constructs. The Rab8a *wildtype* (WT) construct was made by site directed mutagenesis using the following primers: 5’-GG GAT ACC GCG GGT CAG GAA CGT TTT CGT AC-3’ (for) and: 5’-GT ACG AAA ACG TTC CTG ACC CGC GGT ATC CC-3’ (rev). Expression was carried out in 2xYT Broth supplemented with 34 μg/ml kanamycin (FORMEDIUM™) at 37°C. At an OD_{600} of 0.7 the culture was induced with 0.5 mM IPTG (FORMEDIUM™), after which cells were grown for a further 4 hours at 37°C or 18°C overnight. Cells were harvested by centrifugation and the pellets were resuspended in His-tag extraction buffer (20 mM Tris-HCl, 300 mM NaCl, 5 mM $MgCl_2$, 20 mM imidazole and 10 mM β-mercaptoethanol, pH 8.0) along with 0.5 mM PMSF protease inhibitor (Sigma). Cells were lysed by sonication and the cell lysate was centrifuged at 26,000 x *g* for 45 minutes at 4°C to remove cellular debris. The supernatants were filtered and loaded onto a nickel agarose resin (QIAGEN). The resin was washed with a 10-fold excess of extraction buffer and 5-fold excess wash buffer (extraction buffer supplemented with 40 mM Imidazole). The protein was eluted using extraction buffer supplemented with 200 mM imidazole.

Removal of the His_6-tag (Rab8a) was performed by overnight incubation at 4°C with thrombin (GE Healthcare), followed by a second Ni^{2+}-agarose column. The ‘flow-through’ fractions were collected, while the uncut proteins remained on the resin. Soluble aggregates were eliminated by running the sample through a Superdex 75 (16/60) gel filtration column (GE Healthcare) equilibrated in column buffer (20 mM Tris-HCl, 100 mM NaCl, 5 mM $MgCl_2$, 1 mM DTT, pH 7.5). A peptide corresponding to residues 129-165 of RILPL2 was synthesized with an N-terminal hexahistidine tag (His_6-RILPL2, Genscript). The peptide was solubilized in matching buffer with Rab (20 mM Tris-HCl, 100 mM NaCl, 5 mM $MgCl_2$, 1 mM DTT, pH 7.5) prior to crystallization trials and calorimetry.

The cDNA for full-length RILPL2 (1-211) and the GTD of myosin Va (1462-1853), optimized for E.coli expression, were obtained from Genscript. The cDNAs were each synthesized with a 5’ NdeI extension and a 3’ extension comprising 2xSTOP codons (TAA-TGA) followed by a BamH1 site. The cDNA for RILPL2 was subcloned into the Nde/Bam site of pET15b, while the cDNA for

MyoVa(GTD) was subcloned into the identical site in pET28b. Expression and purification of these proteins were performed as described for Rab8a above, except that the polyhistidine tag was not removed from RILPL2 for the pulldown experiment.

Rab8a Nucleotide Exchange

For the pulldown with full-length RILPL2, nucleotide exchange was performed using purified WT Rab8a incubated in 10 mM EDTA for 10 minutes at room temperature in the presence of 10X molar excess GDP. The exchange was terminated by addition of 15 mM $MgCl_2$ and excess nucleotides were removed by running samples through a PD10 column (GE healthcare), or by immediate gel filtration chromatography. To verify successful exchange, 100 μL the protein (>1mg/mL) was boiled for 10 min at 95°C to denature the protein and release the nucleotide, followed by centrifugation for 30 min 16,000 x *g*, 4°C to remove precipitated protein. The supernatant was mixed with running buffer (100 mM potassium phosphate, 8 mM thiobarbituric acid, pH 6.5) at a 1:1 ratio. The samples were loaded on an Acquity Ultra Performance system (Waters Corporation, Milford, MA, USA; or Varian 920 LC machine, Agilent, Stockport, UK) equipped with a ZORBAX 300SB-C18 column (Agilent, Stockport, UK). Elution profiles of GMP, GDP, GTP (Sigma Aldrich) and GppNHp (Jena Bioscience, Germany) were subjected to HPLC and compared with Rab8a. The nucleotide state of Rab8a(Q67L) was confirmed to be GTP-bound using the analytical HPLC strategy.

In Vitro Kinase Assays

It has recently been shown that the MST3 kinase can specifically and efficiently phosphorylate Rab8a at Thr72 *in vitro* (Vieweg et al., 2019). As MST3 is much easier to express than LRRK2, we decided to phosphorylate Rab8a at T72 using recombinant MST3. For comparison of Rab8a phosphorylation by LRRK2 and MST3, kinase assays were performed with shaking at 30°C for 3h with molar concentrations as indicated of 970-end length LRRK2 WT or G2019S (PV4873 and PV4882 respectively, ThermoFisher) or GST-MST3 (supplied by MRC Reagents and Services, DU30889) and 2 μM of Rab8a 1-181 Q67L or Q67L+T72E as a negative control. The kinase reaction buffer is 50 mM Tris-HCl pH 7.5, 10 mM $MgCl_2$, 150 mM NaCl, 2 mM ATP. Efficiency of Rab8a phosphorylation was compared using PhosTag gel electrophoresis and immunoblotting with Rab8a-pT72 antibody (Figure S1).

Phosphorylation of Rab8a

Full length GST-MST3 produced in insect cells (DU30889) was obtained from MRC-PPU Reagents and Services (https://mrcppureagents.dundee.ac.uk/reagents-proteins/overview). Rab8a was incubated with GST-MST3 at molar ratios between 4:1 to 9:1 (substrate:enzyme). Typical concentrations of Rab8a were 1-3 mg/ml, while the concentration of MST3 was 1 mg/ml in a total volume between 2-15 ml. The buffer of the reaction was adjusted to 50 mM Tris-HCl, 150 mM NaCl, 10 mM $MgCl_2$ and 2 mM ATP, pH 7.5. The reaction mixture was incubated at room temperature overnight (12-18 hours). To separate pRab8a from the non-phosphorylated form, the reaction mixture was dialyzed against low-salt ion exchange buffer (10 mM MES, 10 mM NaCl, 5 mM $MgCl_2$, 1 mM DTT, pH 5.2) for two hours and then loaded onto a MonoS 5/50 GL column (GE Healthcare) equilibrated to the low-salt ion-exchange buffer. Elution of pRab8a was performed by running a 50% gradient from low- to high-salt buffer (10 mM MES, 1 M NaCl, 5 mM MgCl2, 1 mM DTT, pH 5.2) over 30 column volumes (Figure S2). The phosphorylation of $Rab8a_{1-181}$ was confirmed by PhosTag gel electrophoresis. In order to stabilize pRab8a, the pH was adjusted to pH 7.5 immediately after elution from the ion-exchange column.

Crystallization, Data Collection and Refinement

Crystals of pRab8(Q67L): His_6-RL2 complex were obtained in a 1:1 molar ratio of protein:peptide at a total of 12 mg/mL. Crystals were grown in 100 mM HEPES buffer (pH 7), 10% PEG4,000, and 10% 2-propanol. Plate-like crystals were harvested in precipitant supplemented with 25% glycerol and stored frozen in liquid nitrogen. X-ray data were collected under a cryogenic nitrogen stream at 100K (beamline 24-ID-C, Advanced Photon Source).

Native diffraction data were reduced using XDS and aimless, followed by structure determination using the Phaser software in the PHENIX package (Adams et al., 2010; McCoy et al., 2007). Initial rounds of molecular replacement using Rab8a [GppNHp, PDB code 4lhw; (Guo et al., 2013)] resulted in a solution for 2 molecules in the asymmetric unit. Following successful identification of Rab8a in the crystal lattice, the electron density for the coiled coil of the effector was apparent. Side chains for RILPL2 were clear in the initial electron density, and refinement was performed using multiple rounds of model building and energy minimization using PHENIX and COOT (Emsley et al., 2010). The asymmetric unit contains two molecules of Rab8a (A:4-176, B:2-176) bound to GTP and a magnesium ion, and two molecules of the effector (C:129-159, E:129-160). The hexahistidine tag at the N-termini of the effector is not seen in electron density maps, except for one histidine at the N-terminus of chain C. Comprehensive validation, including MolProbity, was performed during the refinement process to gauge the quality of the model (Williams et al., 2018). Statistics of the data collection and refinement are shown in Table 1.

Structural Analyses and Superpositions

In general, structures were aligned using the ‘secondary structure matching’ (SSM) protocol in COOT. The backbone superpositions of Rab8a from multiple structures (complexed, uncomplexed) typically aligned with an RMSD of 0.4 Å. The heterotetrameric structures of Rab8a:RILPL2 and Rab7:RILP were aligned using the Superpose software in CCP4 (Krissinel and Henrick, 2004; Winn et al.,

2011). In order to better visualize the relative positions of the effectors, secondary structures from all 4 molecules in each complex were aligned. A total of 330 residues were matched, including 21 residues from each chain of RILP and RILPL2. The overall RMSD for the backbone atoms was approximately 3 Å.

Pulldown Assays, Isothermal Titration Calorimetry, and Static Light Scattering

Calorimetry was performed in triplicate on an ITC-200 instrument (Malvern Panalytical). Protein concentrations were calculated based on their Abs_{280} using a ND-1000 NanoDrop spectrophotometer (Thermo Scientific). Following purification of Rab8a, the protein was dialyzed together in the same buffer as RILPL2 (10 mM Tris-HCl, 300 mM NaCl, 5 mM $MgCl_2$, 20 mM imidazole and 1 mM DTT). Samples were centrifuged at 13,200 rpm for 10 minutes prior to concentration determination and ITC analysis. The concentrations of proteins for injections were between 400-600 μM (His_6-RILPL2, residues 129-165) and 40-60 μM Rab8a and pRab8a (1-181).

For *in vitro* pulldowns, full-length RILPL2 (1-211) and the GTD of myosin Va (residues 1462-1853) were used. Rabs, RILPL2, and MyoVa (GTD) were mixed together in 1.5 mL centrifuge tubes at final concentrations ranging from 2.5-10 μM with 25 μl Ni^{2+}- agarose resin in a final volume of 1ml of binding buffer (20 mM Tris pH 8.0, 300 mM NaCl, 20 mM imidazole, 5 mM $MgCl_2$, 10 mM β-mercapotoethanol). The reaction mixture was subjected to mild shaking for 15 minutes. Following gentle centrifugation (1,000 rpm), the resin was washed 3 times with 1 ml of the binding buffer. Following release of proteins from resin with 50 μl elution buffer (20 mM Tris-Cl pH 8.0, 300 mM NaCl, 200 mM imidazole), samples were subjected to SDS-PAGE and visualization with 0.5% Coomassie Brilliant Blue. Quantification of pulldowns was carried out using the Gel analyzer function of ImageJ(Schindelin et al., 2012), and statistical analyses were done using Microsoft Excel, version 15.28.

Static light scattering coupled to gel filtration was performed using a miniDAWN system (Wyatt Corp), an Optilab rEX refractometer, and a Superdex 200 (10/300) column. Full-length RILPL2 with an uncleaved polyhistidine tag was used, and 500μL was injected at a concentration of 1mg/mL. Data were processed using Astra software version 5.3.

Plasmids for Cellular Assays

The plasmids used for co-immunoprecipitation experiments were acquired from MRC PPU Reagents and Services (https://mrcppureagents.dundee.ac.uk/reagents-proteins/overview): HA-empty pCVM5 (DU49303); GFP-empty pcDNA5 (DU13156); Flag-LRRK2 R1441G pCMV (DU13077); Flag-LRRK2 Y1699C pCMV (DU13165); HA-Rab8a WT pCMV (DU35414); HA-Rab10 WT pCMV (DU44250); RILPL2-GFP WT pcDNA5D FRT/TO (DU27481); RILPL2-GFP R130K pcDNA5D FRT/TO (DU68258), RILPL2-GFP R130A pcDNA5D FRT/TO (DU68022); RILPL2-GFP R130Q pcDNA5D FRT/TO (DU27521); RILPL2-GFP R130E pcDNA5D FRT/TO (DU27520); RILPL2-GFP P131A pcDNA5D FRT/TO (DU68030); RILPL2-GFP P131C pcDNA5D FRT/TO (DU68031); RILPL2-GFP P131K pcDNA5D FRT/TO (DU68256), RILPL2-GFP P131R pcDNA5D FRT/TO (DU68257) RILPL2-GFP R132K pcDNA5D FRT/TO (DU68023); RILPL2-GFP R132A pcDNA5D FRT/TO (DU67110); RILPL2-GFP R132Q pcDNA5D FRT/TO (DU68037); RILPL2-GFP R132E pcDNA5D FRT/TO (DU27522); RILPL2-GFP F133A pcDNA5D FRT/TO (DU68033); RILPL2-GFP L135A pcDNA5D FRT/TO (DU68032); RILPL2-GFP R139A pcDNA5D FRT/TO (DU68025); RILPL2-GFP R139Q pcDNA5D FRT/TO (DU68024); RILPL2-GFP R139E pcDNA5D FRT/TO (DU68026); RILPL2-GFP K149A pcDNA5D FRT/TO (DU68029); RILPL2-GFP K149Q pcDNA5D FRT/TO (DU68027); RILPL2-GFP K149E pcDNA5D FRT/TO (DU68028); RILPL2-GFP E157A pcDNA5D FRT/TO (DU68036); RILPL2-GFP E157Q pcDNA5D FRT/TO (DU68034); RILPL2-GFP E157K pcDNA5D FRT/TO (DU68035), HA-Rab8a Q67L pCMV (DU39393), HA-Rab8a T22N pCMV (DU39392), HA-Rab8a T4A pCMV5 DU68045), HA-Rab8a D44A pCMV5 (DU68041), HA-Rab8a D44N pCMV5 (DU68039), HA-Rab8a D44K pCMV5 (DU68040), HA-Rab8a K58A pCMV5 (DU68044), HA-Rab8a K58Q pCMV5 (DU68042), HA-Rab8a K58E (DU68043), JIP3-GFP pcDNA5D FRT/TO (DU27721), JIP4-GFP pcDNA5D FRT/TO (DU27684), RILPL1-GFP pcDNA5D FRT/TO (DU27305).

Antibody Reagents

Antibodies used in this study were diluted in 5% w/v bovine serum albumin in TBS supplemented with 0.1% Tween-20 and 0.03% w/v sodium azide. The Rabbit monoclonal antibody for total LRRK2 (N-terminus) was purified at the University of Dundee (Dzamko et al., 2012). Anti-GFP (PABG1, Chromotek, used at 1:1000) anti-GFP (#2956, CST, used at 1:1000), anti-HA (3F10, Merck, used at 1:1000), anti-pT72-Rab8a (MJF-R20, Abcam, used at 0.5 μg/mL), anti-LRRK2 C-terminal (N241A/34, Neuromab, used at 1:1000), and anti-αTubulin (3873S, CST, used at 1:5000), anti-GAPDH (#sc-32233, Santa Cruz Biotechnology, used at 1:5000). Secondary antibodies used were Licor IRDye for 800CW goat anti-rabbit (925-32211), goat anti-mouse (926-32210) and 680LT goat anti-rat (925-68029) and goat anti-mouse (926-68020), all used at 1:10,000 dilution in TBS with 0.1% v/v Tween-20 (TBS-T) and horseradish peroxidase-conjugated rat IgG secondary antibody (#31470, Thermo Fisher Scientific) used at 1:10,000 dilution in 5% non-fat dry milk dissolved in TBS-T.

Culture and Transfection of Cells

HEK293 cells were cultured in Dulbecco's modified Eagle medium (Glutamax, Gibco) supplemented with 10% fetal bovine serum (FBS, Sigma), 100 U/ml penicillin and 100 μg/ml streptomycin. Transient transfections were performed 40-48 hr prior to cell lysis using polyethylenimine PEI (Polysciences) at around 60-70% confluence. Transfections for co-immunoprecipitation experiments were done in 10 cm round cell culture dishes using 3 μg of Flag-LRRK2 R1441G or Flag-LRRK2 Y1699C as indicated, 1 μg of HA control or HA-Rab8a or HA-Rab10 and 1 μg of GFP control, RILPL2-GFP or JIP3/4-GFP cDNA construct per dish diluted in 1 mL of OPTIMEM

media and supplemented with 20 μg of PEI and incubated for 20 min before being added to the cell media. 1 h before lysis cells were treated with 500 nM of MLI-2 inhibitor or 0.1% DMSO control. Lysates were clarified by centrifugation at 17,000 x *g* for 10 min.

Co-Immunoprecipitation of Rab GTPases and RILPL2, JIP3 and JIP4

Cells were washed with PBS and lysed in lysis buffer - 50 mM Tris-HCl pH 7.5, 1 mM EGTA, 10 mM sodium β-glycerophosphate, 50 mM sodium fluoride, 5 mM sodium pyrophosphate*10H_2O, 0.27 M sucrose and supplemented fresh before lysis with 1% v/v Triton-x100, 1 tablet of cOmplete Mini (EDTA-free) protease inhibitor (Merck, 11836170001) per 10 mL of buffer, 0.1 μg/mL of microcystin and 1 μM of sodium orthovanadate.

For GFP immunoprecipitation, lysates were incubated with nanobody αGFP binder sepharose from MRC PPU Reagents and Services for 1 hr (15 μl of packed resin/0.5 mg of lysate). Bound complexes were recovered by washing the beads three times with wash buffer (50 mM Tris-HCl pH 7.5, 150 mM NaCl) before eluting with 2xSDS/PAGE sample buffer supplemented with 1% v/v 2-mercaptoethanol. The samples were denatured at 70°C for 10 min and the resin was separated from the sample by centrifugation through a 0.22 μm Spinex column (CLS8161, Sigma).

Gel Electrophoresis and Immunoblot Analysis

Samples were run on gels consisting of a 4% w/v acrylamide stacking gel [4% w/v acrylamide, 0.125 M Tris-HCl pH 6.8, 0.2% v/v Tetramethylethylenediamine (TEMED) and 0.08% w/v ammonium persulphate (APS)] and 10% w/v acrylamide separating gel [10% w/v acrylamide, 0.375 M Bis-Tris pH 6.8, 1% v/v tetramethylethylenediamine (TEMED) and 0.05% w/v ammonium persulphate (APS)] in MOPS buffer (50 mM MOPS, 50 mM Tris, 1 mM EDTA, 0.1% w/v SDS) at 90-120 V. For Coomassie staining, gels were stained with InstantBlue™ Ultrafast Protein Stain (ISB1L, Sigma-Aldrich) according to the manufacturer's instructions and the gels were imaged using LICOR Odyssey CLx. For immunoblot analysis, proteins were electrophoretically transferred onto nitrocellulose membranes (Amersham Protran 0.45 μm NC; GE Healthcare) at 90 V for 90 min on ice in transfer buffer [48 mM Tris/HCl, 39 mM glycine, 20% v/v methanol]. Transferred membranes were blocked with 5% w/v non-fat dry milk dissolved in TBS-T [20 mM Tris/HCl, pH 7.5, 150 mM NaCl and 0.1% v/v Tween 20] at room temperature for 1 h. Membranes were then incubated with primary antibodies overnight at 4°C. After washing membranes in TBS-T 3x15 min, membranes were incubated with secondary antibodies at room temperature for 1 h. After washing membranes in TBS-T 3x15 min membranes were scanned using LICOR Odyssey CLx.

PhosTag Gel Electrophoresis and Immunoblot Analysis

Samples were supplemented with 10 mM $MnCl_2$ before loading gels. Gels for Phos-tag SDS/PAGE consisted of a stacking gel [4% w/v acrylamide, 0.125 M Tris/HCl, pH 6.8, 0.2% v/v tetramethylethylenediamine (TEMED) and 0.08% w/v ammonium persulfate APS] and a separating gel [10% w/v acrylamide, 375 mM Tris/HCl, pH 8.8, 75 μM PhosTag reagent (MRC PPU Reagents and Services), 150 μM $MnCl_2$, 0.1% v/v TEMED and 0.05% w/v APS]. After centrifugation at 17,000 x g for 1 min, samples were loaded and electrophoresed at 90 V with the running buffer [25 mM Tris/HCl, 192 mM glycine and 0.1% w/v SDS]. For Coomassie staining, gels were stained with InstantBlue™ Ultrafast Protein Stain (ISB1L, Sigma-Aldrich) according to the manufacturer's instructions and the gels were imaged using LICOR Odyssey CLx. For immunoblot analysis, gels were washed 3x10 min in 48 mM Tris/HCl, 39 mM glycine, 10 mM EDTA and 0.05% w/v SDS followed by one wash in 48 mM Tris/HCl, 39 mM glycine and 0.05% w/v SDS for 10 min. Proteins were electrophoretically transferred onto nitrocellulose membranes (Amersham Protran 0.45 μm NC; GE Healthcare) at 100 V for 180 min on ice in transfer buffer [48 mM Tris/HCl, 39 mM glycine, 20% v/v methanol]. Transferred membranes were blocked with 5% w/v non-fat dry milk dissolved in TBS-T [20 mM Tris/HCl, pH 7.5, 150 mM NaCl and 0.1% v/v Tween 20] at room temperature for 1 h. Membranes were then incubated with primary antibodies overnight at 4°C. After washing membranes in TBS-T 3x15 min, membranes were incubated with horseradish peroxidase labelled secondary antibody diluted in 5% skimmed milk powder in TBS-T at room temperature for 1 h. After washing membranes in TBS-T (5x10 mins), protein bands were detected by exposing films (Amersham Hyperfilm ECL, GE Healthcare) to the membranes using an ECL solution (SuperSignal West Dura Extended Duration, Thermo Fisher Scientific).

DATA AND CODE AVAILABILITY

The coordinates for the structure of the pRab8a:RILPL2 complex have been deposited in the Protein Data Bank with accession code 6RIR. PDB codes 4LHW, 4KP3, and 1YHN were referenced in this study.

Structure, Volume *28*

Supplemental Information

Structural Basis for Rab8a Recruitment

of RILPL2 via LRRK2 Phosphorylation of Switch 2

Dieter Waschbüsch, Elena Purlyte, Prosenjit Pal, Emma McGrath, Dario R. Alessi, and Amir R. Khan

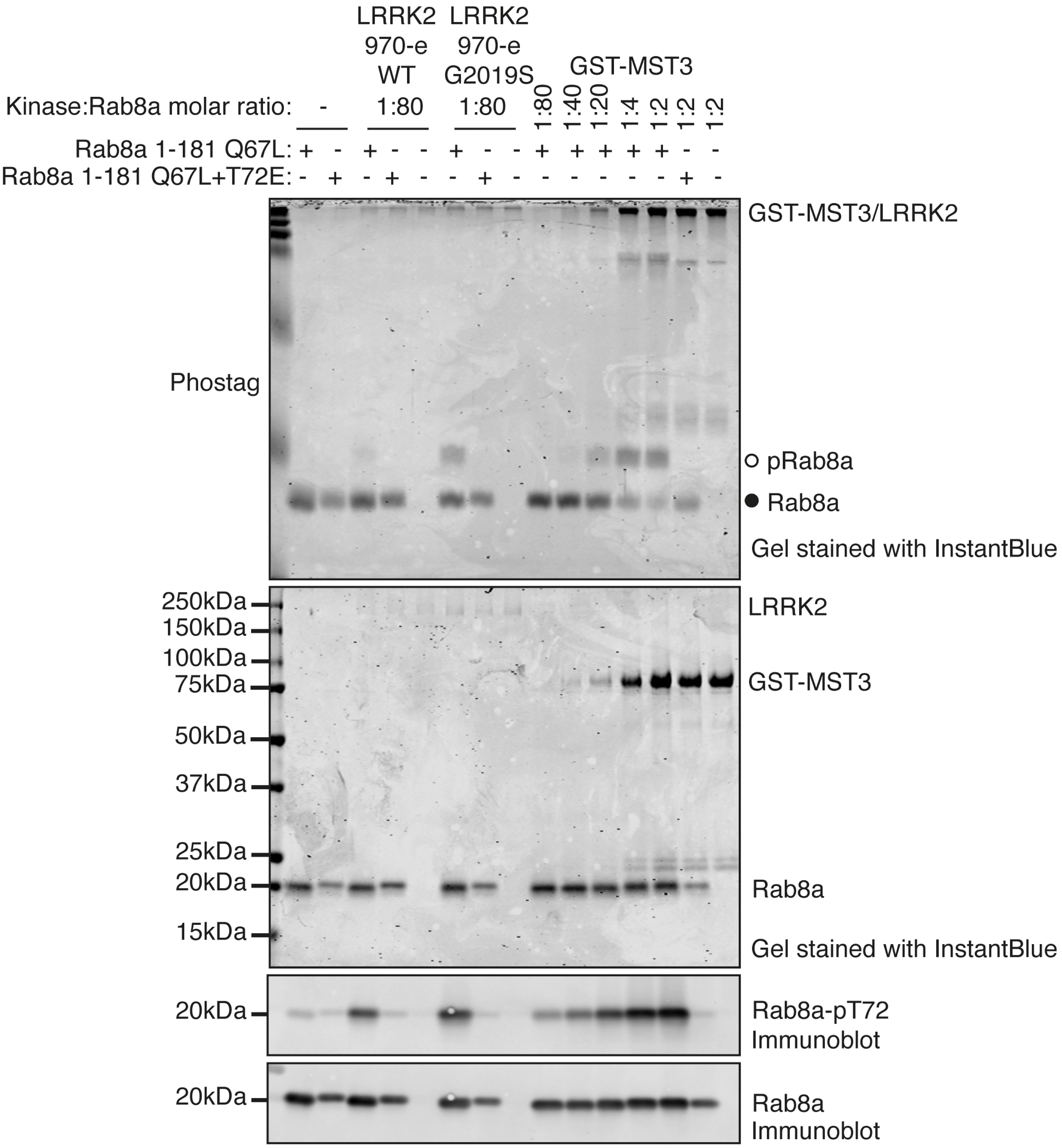

Figure S1, related to Figure 1: Phos-tag analysis of MST3 mediated Rab8a phosphorylation. Side by side analysis of wild type and G2019S insect cell expressed LRRK2 (residue 970-end) and full length insect cell expressed GST-MST3 mediated phosphorylation of recombinant Rab8a[Q67L, residues 1 to 181] or Rab8a[Q67L+T72E, residues 1 to 181]. Reactions were undertaken in the presence of 10 mM MgCl2 and 2 mM ATP for 3 hours at 37oC. Reactions were terminated by the addition of SDS sample buffer. Upper panel an aliquot of the reaction was analyzed by Phos-tag gel electrophoresis and protein visualized Coomassie Blue-staining. Bands corresponding to phosphorylated and non-phosphorylated Rab8a were marked with open (○) and closed (●) circles respectively. Lower panels samples were subjected to conventional gel electrophoresis and either stained with Coomassie (second panel) or subjected to immunoblot analysis using the LI-COR Odyssey CLx Western Blot imaging system with the indicated antibodies at 0.5-1 μg/mL concentration (two bottom panels). Similar results were obtained in at least two separate experiments.

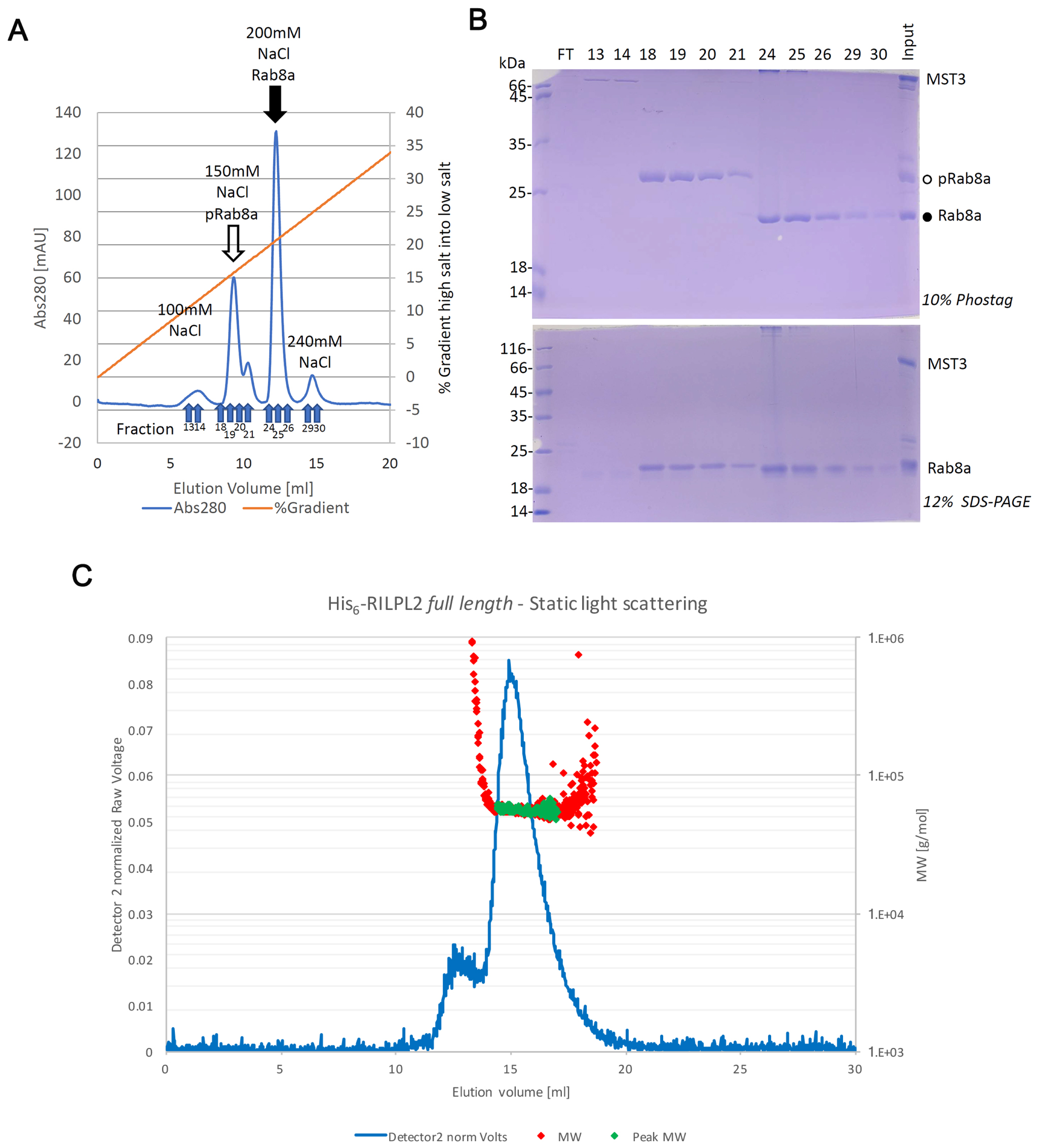

Figure S2, related to Figure 2: Purification of phosphorylated Rab8a. Following incubation of several mg of Rab8a1-181Q67L in the presence of GST-MST3 and ATP, phosphorylated Rab8a (pRab8a) was separated from the unphosphorylated protein by ion-exchange chromatography. A, Following dialysis in low salt buffer, reactants were loaded onto Mono-S cation exchange column (GE Healthcare) and eluted with a 50% gradient from low salt (10 mM) to high salt (1 M) buffered solution. During the salt gradient, 0.5 ml fractions were collected, and the two main peaks (arrows) corresponded to pRab8a and Rab8a. B, PhosTag gel (upper panel) and SDS-PAGE (lower panel) of the collected fractions. FT: unbound fraction flow through; Input: endpoint of phosphorylation reaction before loading the column. The PhosTag gel shows that the peak coming off at 150 mM NaCl contains the phosphorylated Rab8a. C, Static light scattering (SLS) analysis of full-length RILPL2. The monomer, which includes the polyhistidine tag, is 27kDa. The effector runs as a dimer in this experiment (55kDa) with the SLS instrument coupled to a Superdex 200 (10/300) gel filtration column. Detector 2 correspond to the refractive index, which is related to the protein concentration.

A

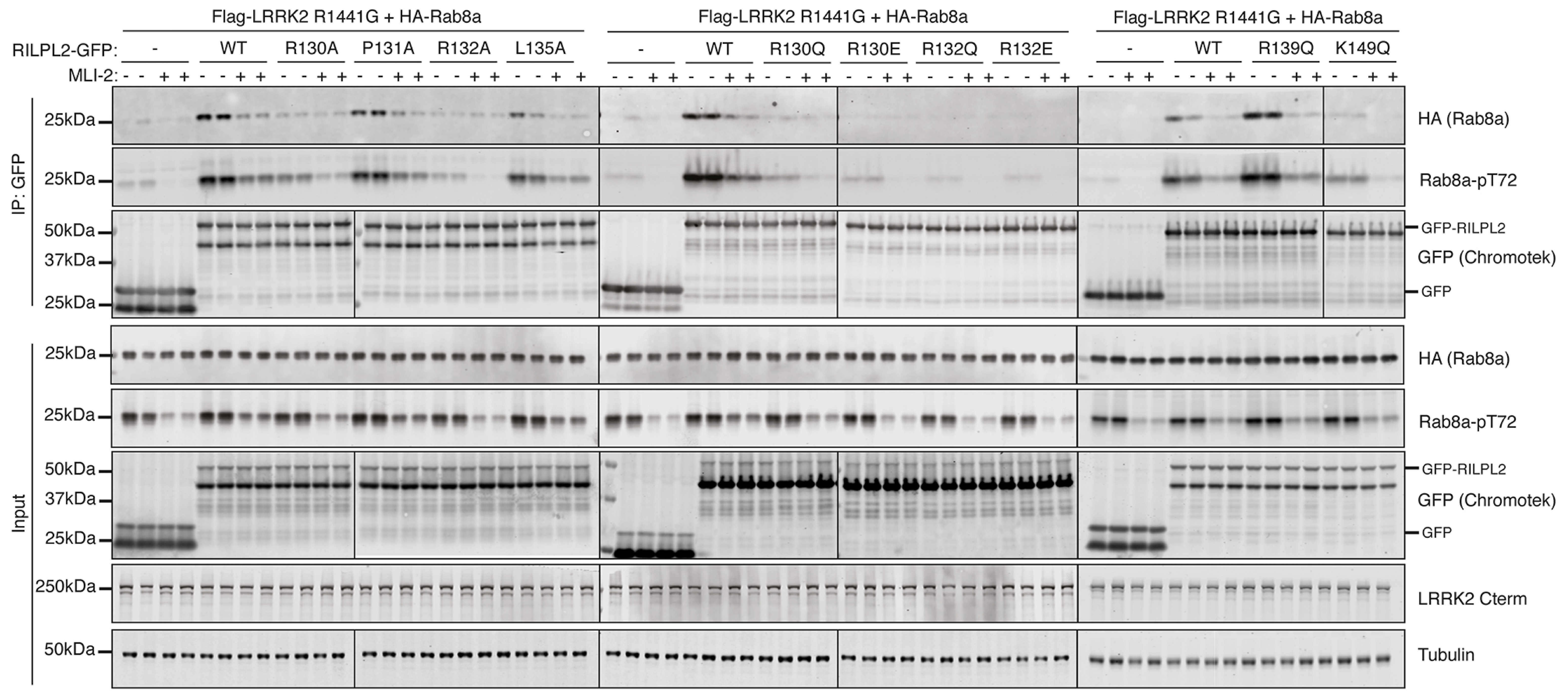

B

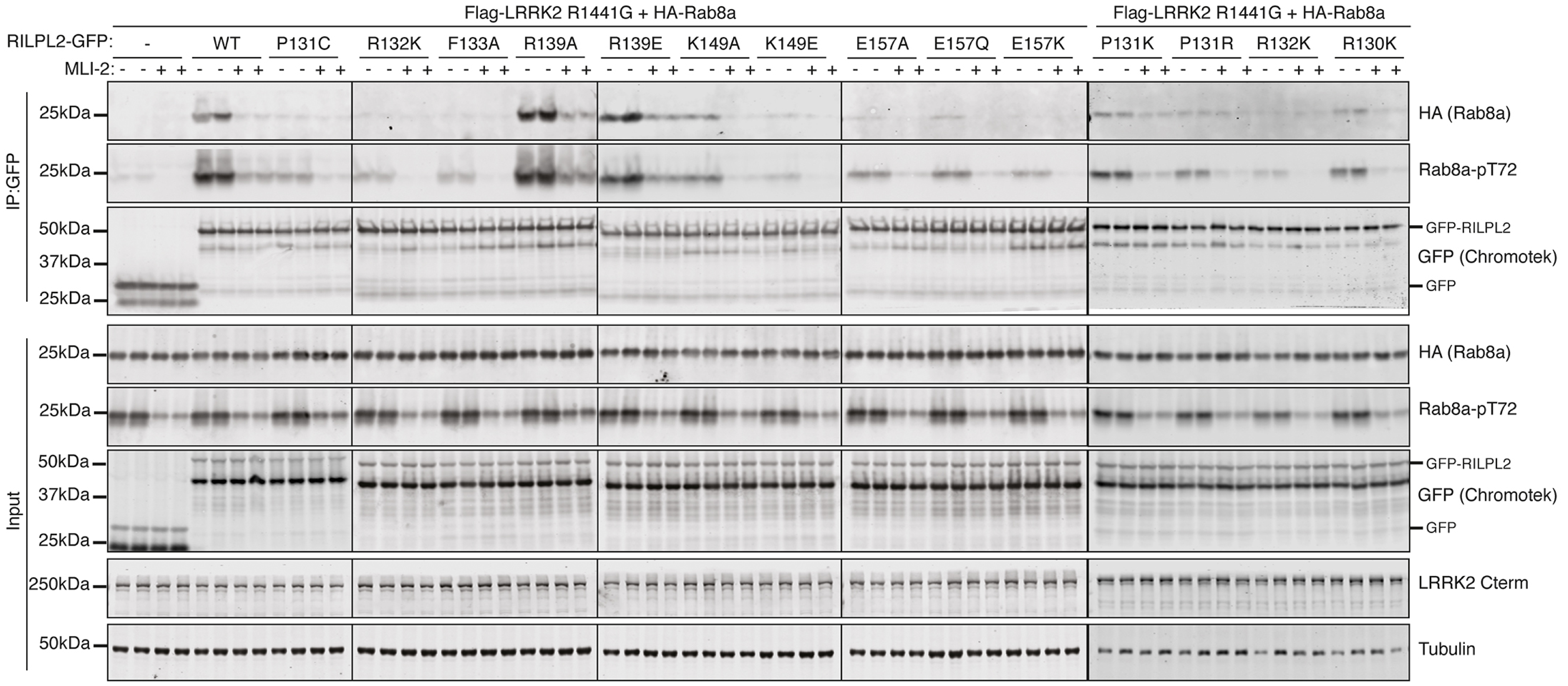

Figure S3, related to Figure 3: Figure legend same as for Figure 3.

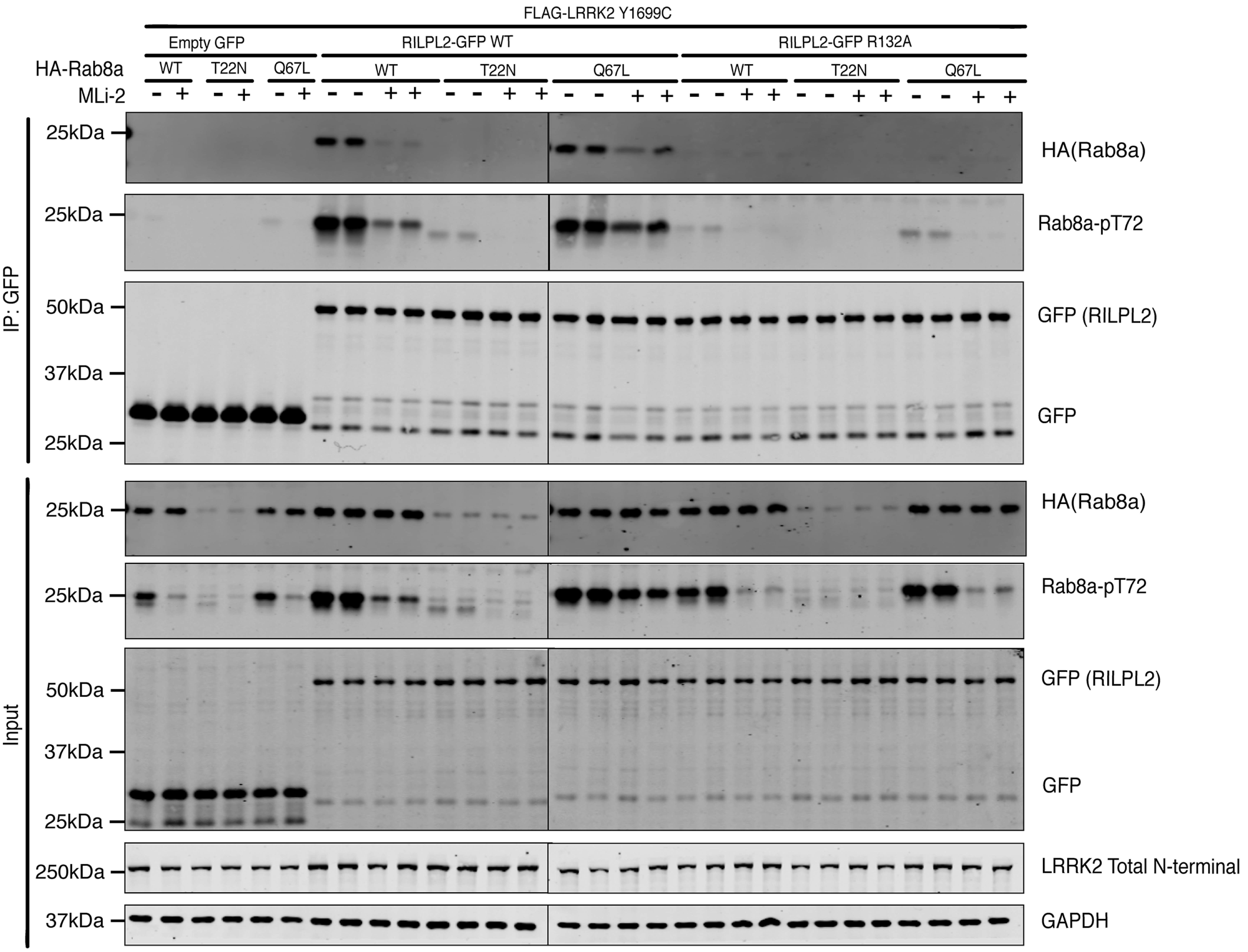

Figure S4, related to Figure 4: Same figure legend as Fig 4B

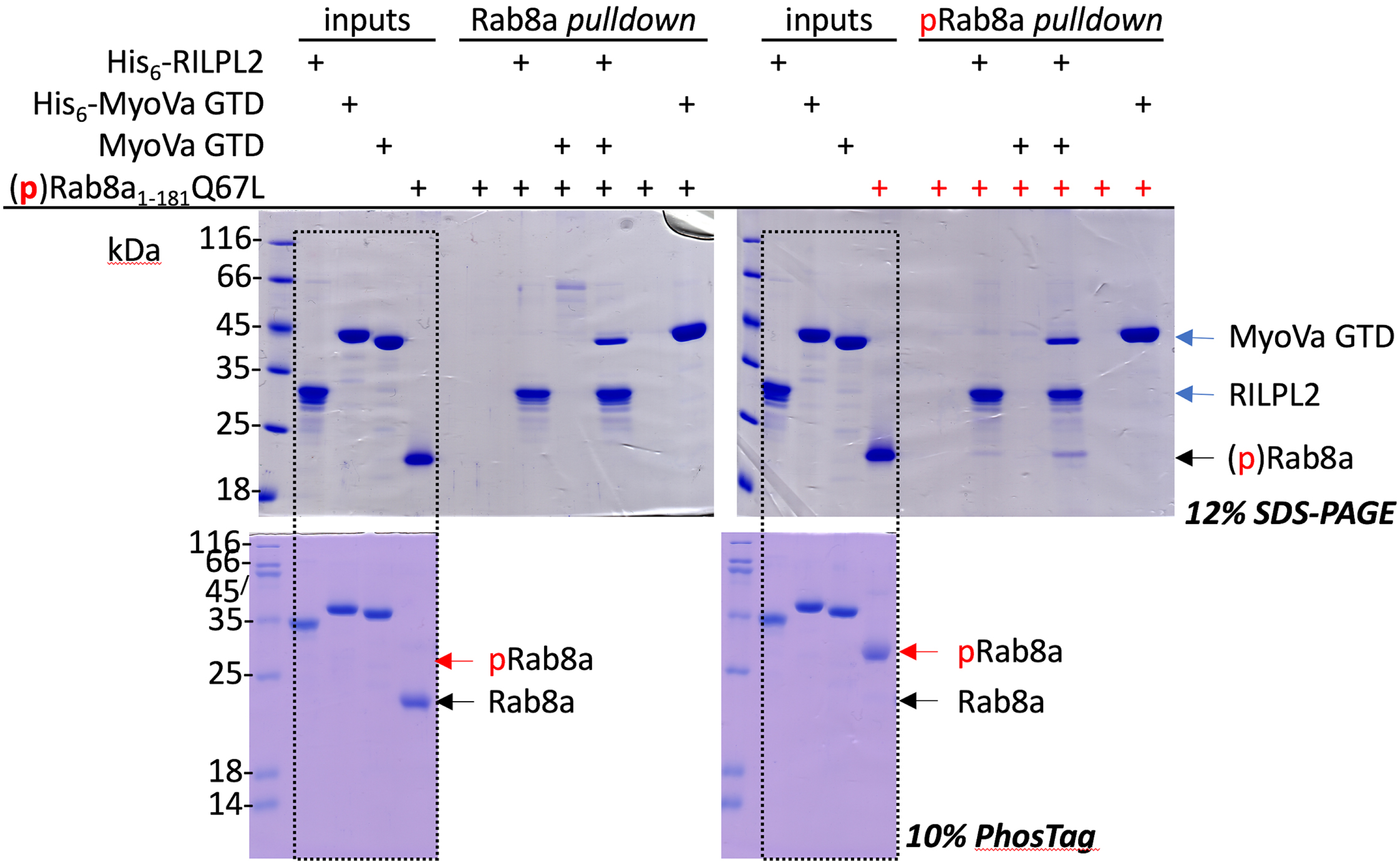

Figure S5, related to Figure 5A: Ni-NTA pulldowns of His6 tagged RILPL2 were performed using 2.5μM concentrations of bait and prey proteins. A control using pRab8a (red) and Rab8a pulldown using His6-tagged GTD of MyoVa confirms that there is no direct binding of either pRab8a or Rab8a to the GTD. Phostag gels of the input proteins are below the pulldowns.

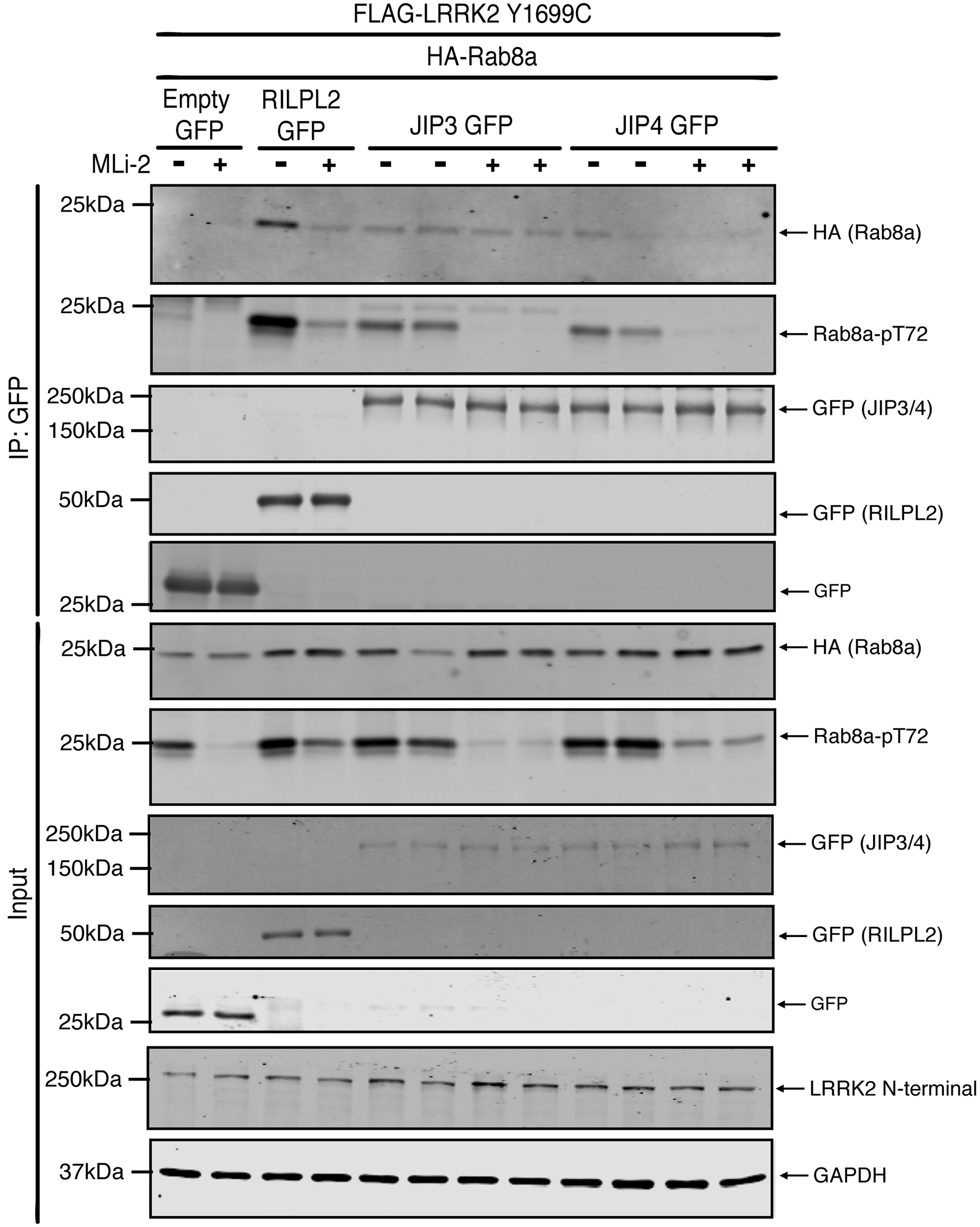

Figure S6, related to Figure 6A: JIP3 and JIP4 bind weakly to both Rab8a and LRRK2-phosphorylated Rab8a in cells. Same figure legend as Fig 6A.
